# Supplementary material for: DPP8/9 processing of human AK2 unmasks an IAP binding motif
Source: EMBO Rep. 2025 May 1;26(11):2819–35. doi: 10.1038/s44319-025-00455-z (PMC12152192; doi:10.1038/s44319-025-00455-z)
Supplement: Supplementary file 1 — Appendix [file 44319_2025_455_MOESM1_ESM.pdf]

APPENDIX

DPP8/9 processing of human AK2 unmasks an IAP binding motif

Kim J. Lapacz[1,#], Konstantin Weiss[1,#], Franziska Mueller[2,3,#], Yuxing Xue[3], Simon Poepsel[4,5], Matthias Weith[1], Tanja Bange[3,*], Jan Riemer[1,5,*]

1, Institute for Biochemistry, University of Cologne, 50674 Cologne, Germany

2, Department of Mechanistic Cell Biology, Max Planck Institute of Molecular Physiology, 44227 Dortmund

3, Institute of Medical Psychology and Biomedical Center (BMC), Faculty of Medicine, LMU Munich, 80336 Munich, Germany

4, Center for Molecular Medicine Cologne (CMMC), Faculty of Medicine and University Hospital, University of Cologne, D-50931 Cologne, Germany.

5, Cologne Excellence Cluster on Cellular Stress Responses in Aging-Associated Diseases (CECAD), University of Cologne, 50931 Cologne, Germany.

#, these authors contributed equally

* address correspondence to

J.R.: jan.riemer@uni-koeln.de, +49-221-470-7306, ORCID 0000-0002-7574-8457

T.B.: tanja.bange@med.uni-muenchen.de, +49-89-218075654, ORCID 0000-0002-9680-8586

Condensed title: DPP8 and DPP9 unmask IAP binding motifs

TABLE OF CONTENT

APPENDIX FIGURES

Appendix Figure S1	page 3
Appendix Figure S2	page 7
Appendix Figure S3	page 8
Appendix Figure S4	page 9
Appendix Figure S5	page 10
Appendix Figure S6	page 11
Appendix Figure S7	page 12
Appendix Figure S8	page 13

APPENDIX REFERENCES page 18

APPENDIX FIGURES

A **SVPAAEPEYPK2^{+} (PEP 4.51E^{-05}) - DDP8/9 cleaved N-terminus in HEK WT**

20240425_TB_SA_WTprot_2.raw (33.69 min)

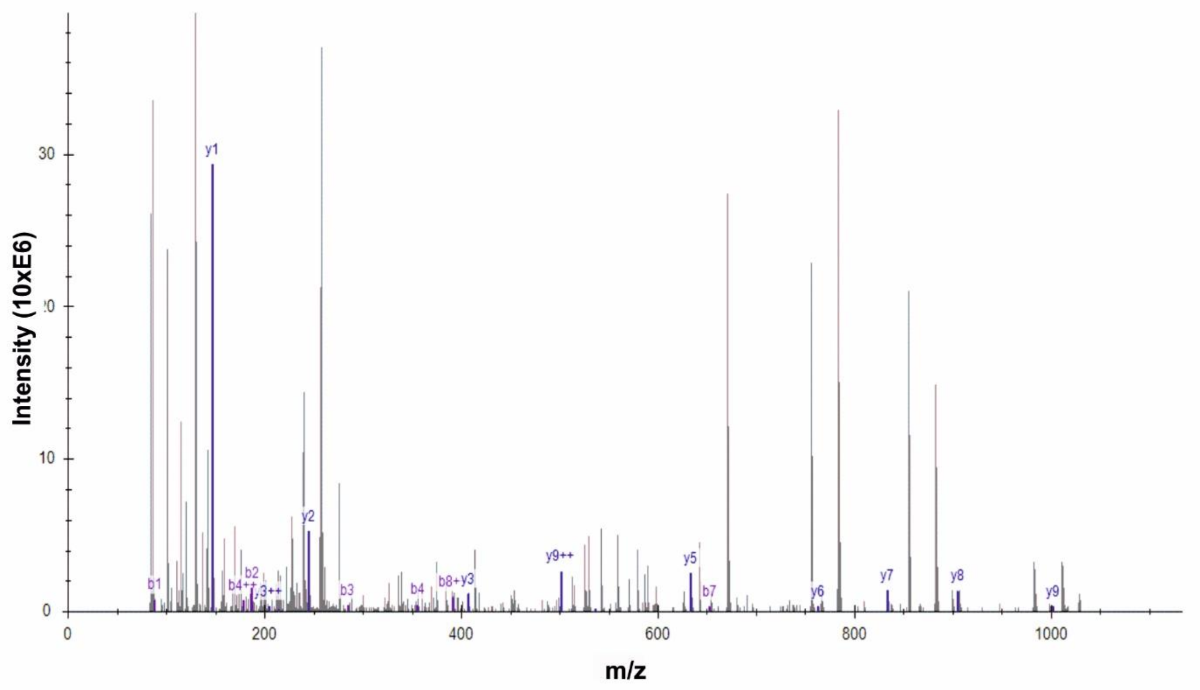

B **SVPAAEPEYPK2^{+} (PEP 2.75E^{-07}) - DPP8/9 cleaved N-terminus in HEK DPP9 KO**

2024_TB_SA_KOprot_2.raw (33,76min)

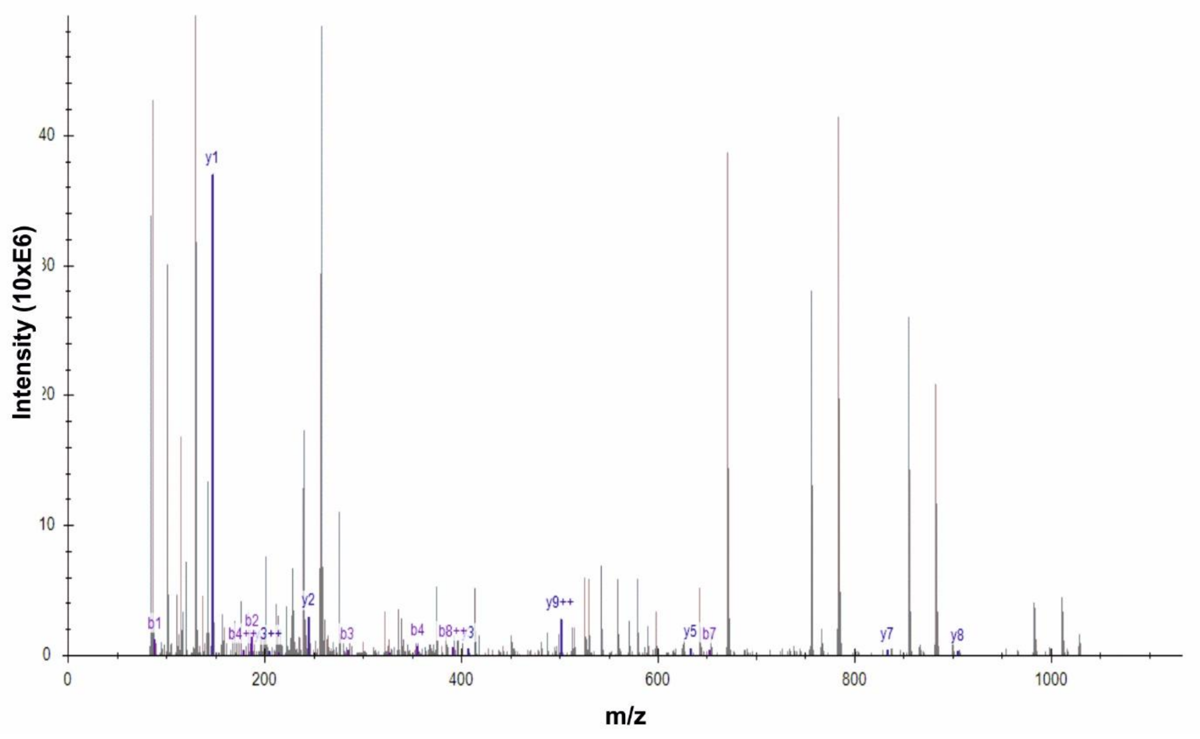

C

APSVPAAEPEYPK2⁺ (PEP 8.24E⁻⁰⁵) - MetAP cleaved N-terminus in HEK WT

20240425_TB_SA_WTprot_2.raw (41.75 min)

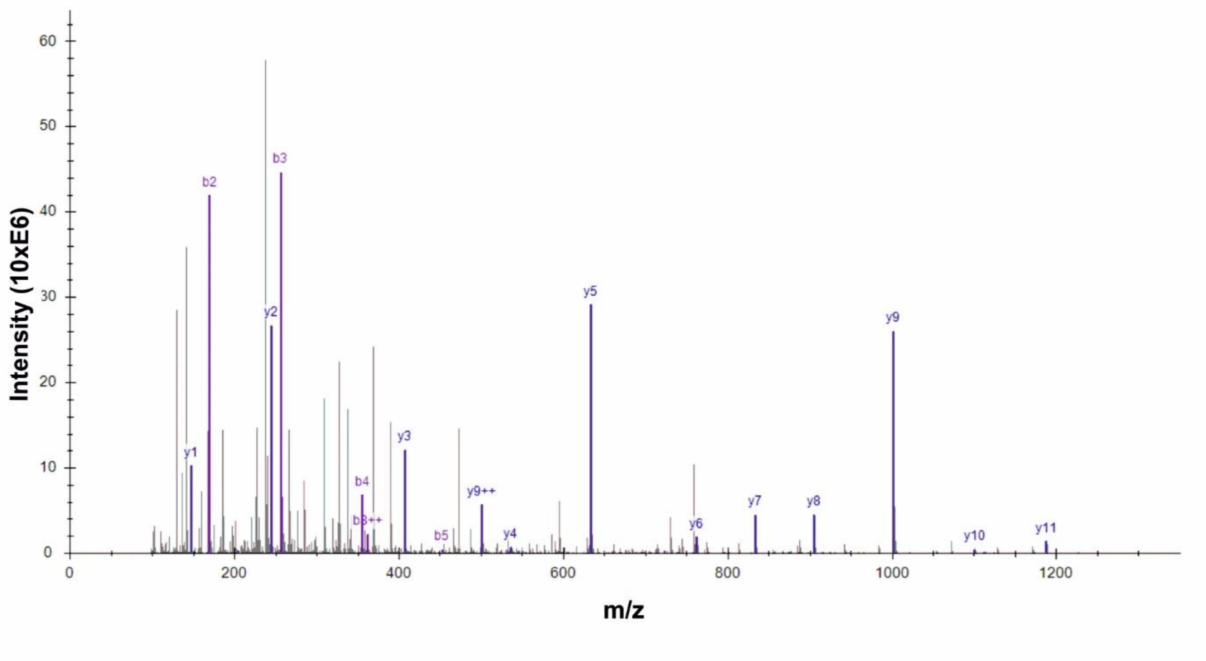

D

APSVPAAEPEYPK2⁺ (PEP 5.15E⁻⁰⁵) - MetAP cleaved N-terminus in HEK DPP9 KO

2024_TB_SA_KOprot_2.raw (41.58min)

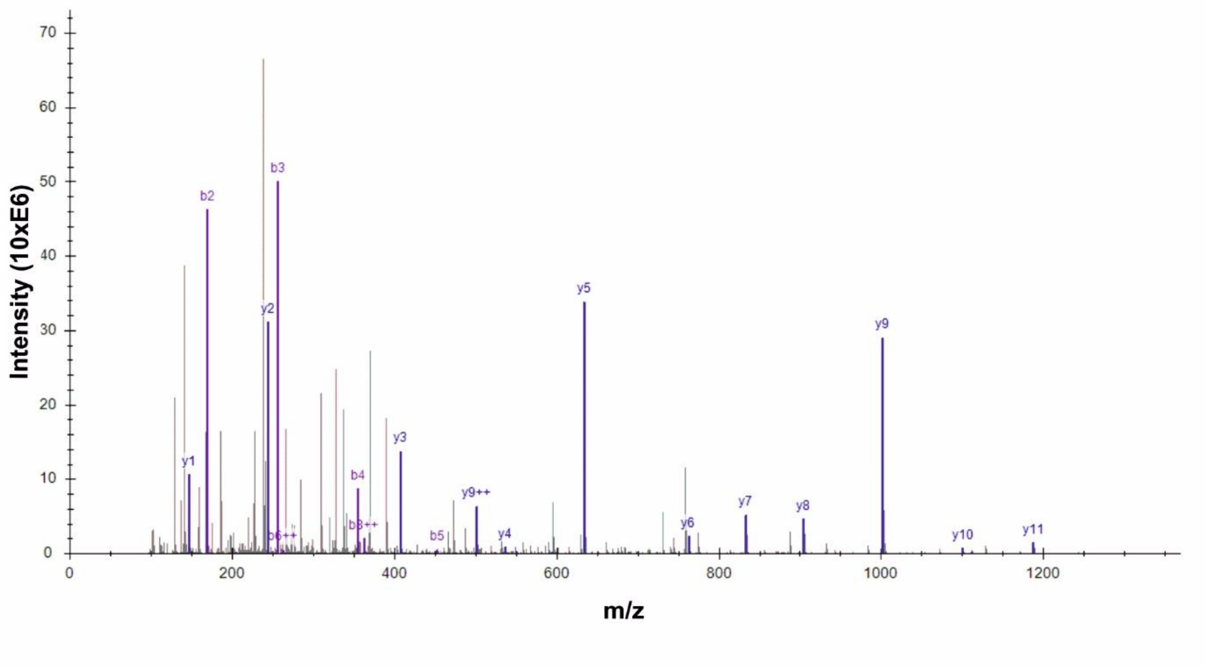

E **M(ac)APSVPAAEPEYPK2⁺ (PEP 8.9E⁻⁰⁵) - unprocessed N-terminus in HEK WT**

20240425_TB_SA_WTprot_3.raw (64.18 min)

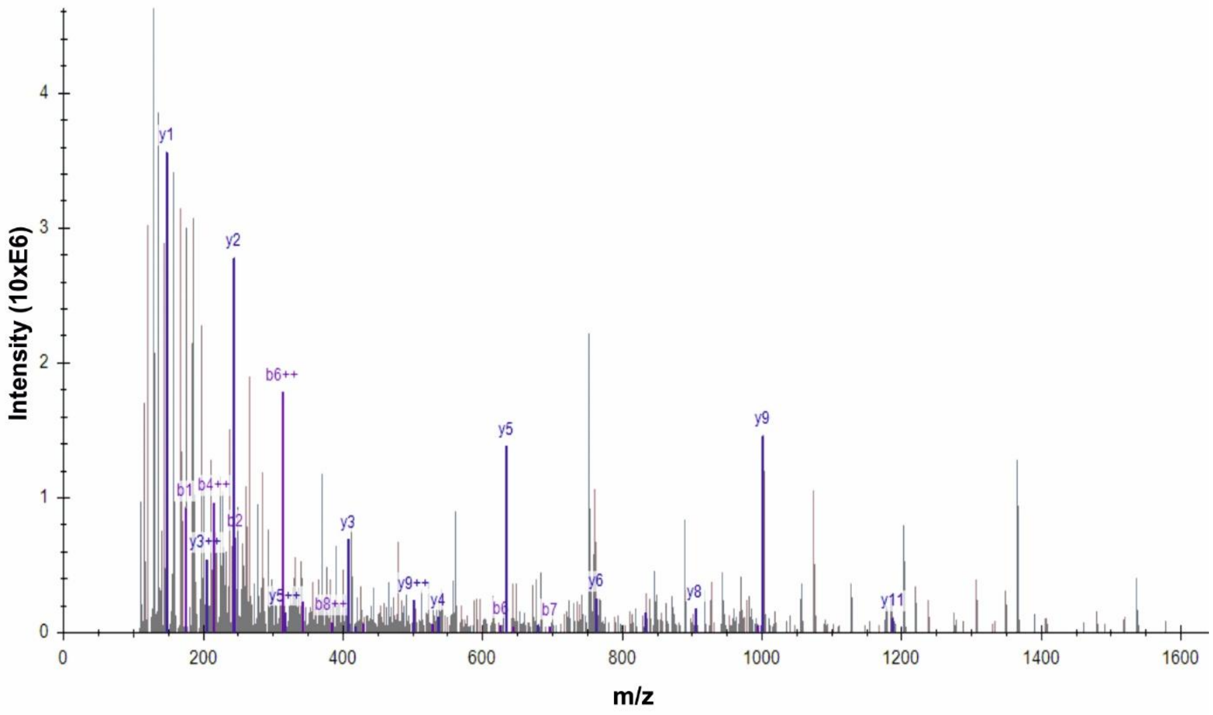

F **M(ac)APSVPAAEPEYPK2⁺ (PEP 0.0007) - unprocessed N-terminus in HEK DPP9 KO**

2024_TB_SA_KOprot_3.raw (64.21min)

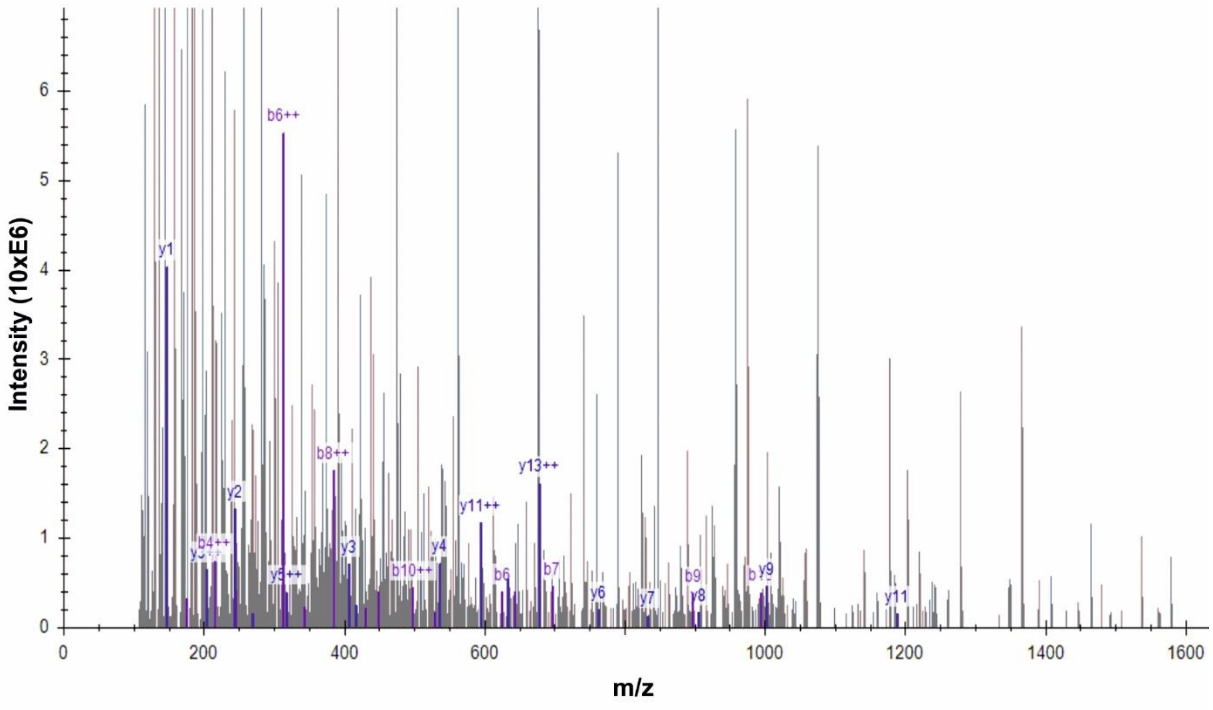

Appendix Figure S1 (related to Figure 1F). MS2 spectra of AK2 N-terminal peptides identified from whole cellular lysates of HEK293 WT and DPP9 KO cells.

A,B. AK2 N-terminal peptide NH_2-SVPAAEPEYPK in WT (A) and DPP9 KO (B) lysate.

C,D. AK2 N-terminal peptide NH_2-APSVPAAEPEYPK in WT (C) and DPP9 KO (D) lysate.

E,F. AK2 N-terminal peptide ac-NH_2-MAPSVPAAEPEYPK in WT (E) and DPP9 KO (F) lysate.

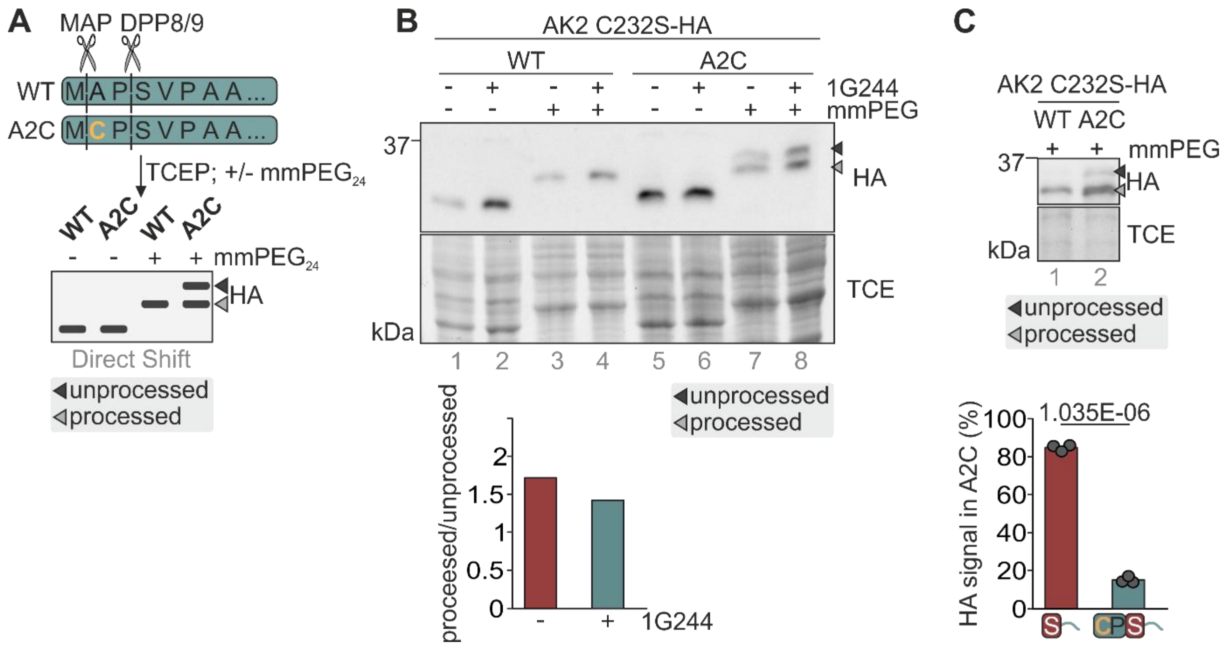

Appendix Figure S2 (related to Figure 1). DPP9 processed the majority of cellular AK2.

A. Visualization of processing events by DPP8/9 using a maleimide shift assay. The AK2 A2C variant allows monitoring the AK2 processing by DPP8/9. Processing will remove the cysteine residue at position 2. If processing does not take place the cysteine can be modified using the maleimide mmPEG24. This will result in a size shift detectable on SDS-PAGE (indicated by the dark arrow).

B. 1G244 stabilizes AK2 variants and minimally shifts processing ratio. Stable inducible cell lines expressing either AK2 C232S or AK2 A2C, C232S were after incubation with or without 1G244 lysed and reduced using TCEP. Then $mmPEG_{24}$ was added to the lysates to modify free cysteines. Samples were analyzed by SDS-PAGE and immunoblot. n=1 biological replicates

C. The majority of AK2 is processed by DPP8/9. Stable inducible cell lines expressing either AK2 C232S or AK2 A2C, C232S were lysed and reduced using TCEP. Then $mmPEG_{24}$ was added to the lysates to modify free cysteines. Samples were analyzed by SDS-PAGE and immunoblot. n=3 biological replicates

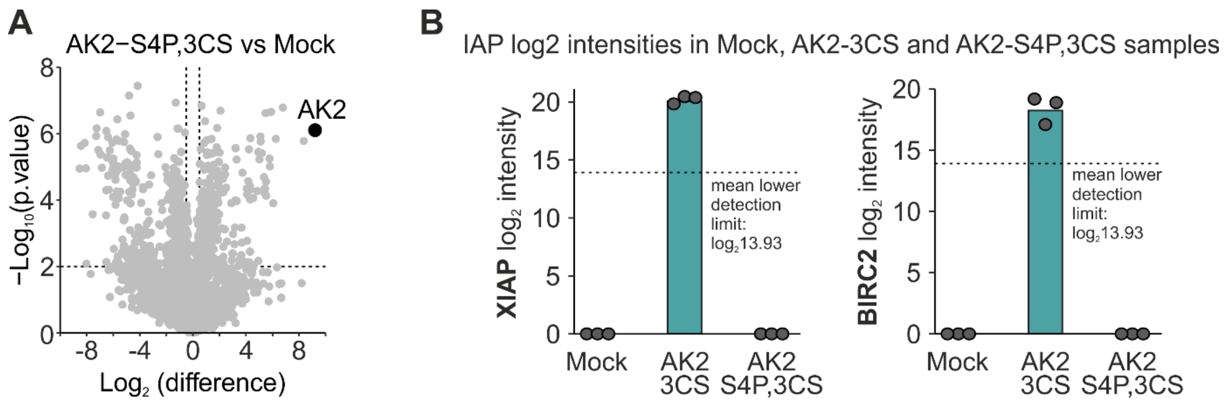

Appendix Figure S3 (related to Figure 2M,N). The IAPs XIAP and BIRC2 can bind to AK2 but not to the S4P mutant of AK2.

A. Neither the terminal mutation of DPP8/9 processed AK2 (S4P) nor the mock control bind to XIAP and BIRC2. AK2 S4P, C40,42,92S-HA (S4P,3CS) and mock treated cells were immunoprecipitated and binding partners were quantified by MS. 2,623 proteins were quantified, number of biological replicates n=3. Data were imputated at the lower end of the distribution (downshift: 2; width:0.3). AK2 and IAPs are highlighted.

B. XIAP and BIRC2 were identified as interaction partners only in cytosolic WT AK2. Measured log_2 intensities of coprecipitates of mock, AK2 C40,42,92S-HA (3CS) and AK2 S4P, C40,42,92S-HA (S4P,3CS) for XIAP and BIRC2 respectively (n=3 biological replicates). Data from **Fig. 2M,N** and **S3A** before data imputation. The lowest measured value for quantified proteins in this data set is inserted as a dashed line.

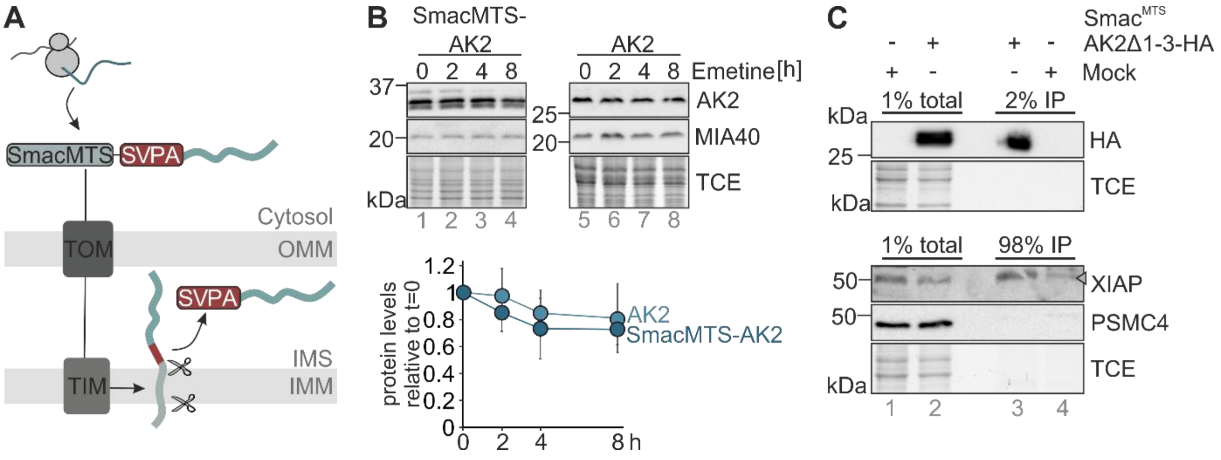

Appendix Figure S4 (related to Figure 2). The IAP XIAP can bind to AK2.

A. Smac^{MTS}-AK2 Δ1-3 is translated in the cytosol. It is then imported into mitochondria where the MTS is removed leaving soluble AK2 exposing its IBM.

B. AK2 with and without Smac^{MTS} is stable in the IMS over time. Stable inducible cell lines expressing AK2 with or without the Smac^{MTS} were tested in an Emetine chase for the indicated amount of time. Samples were lysed and analyzed by SDS-PAGE and immunoblot. n=3 biological replicates.

C. The IAP XIAP can bind to AK2. Stable inducible HeLa cell lines in an AK2 KO background were lysed. Smac^{MTS}-AK2 Δ1-3-HA was precipitated using HA-beads and the resulting precipitate was analysed for XIAP. XIAP was coprecipitated with AK2 indicating interaction of both proteins after cell lysis. Analyses was done by SDS-PAGE and immunoblot. n=3 biological replicates.

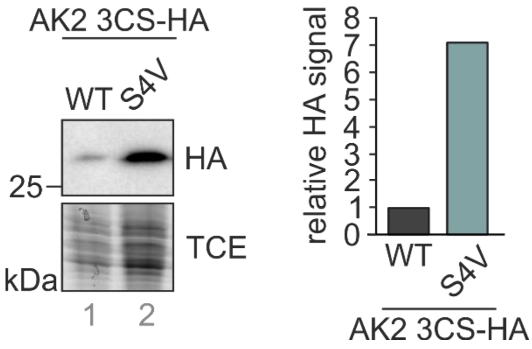

Appendix Figure S5 (related to Figure 3). Mutation of the AK2 IBM stabilizes the protein.

Stable inducible cell lines expressing either the WT or the S4V variant of AK2 were lysed and analyzed via SDS-PAGE and immunoblot. n=4 biological replicates; one replicate is shown, the three other replicates were used in (Finger *et al.*, 2020) for quantification.

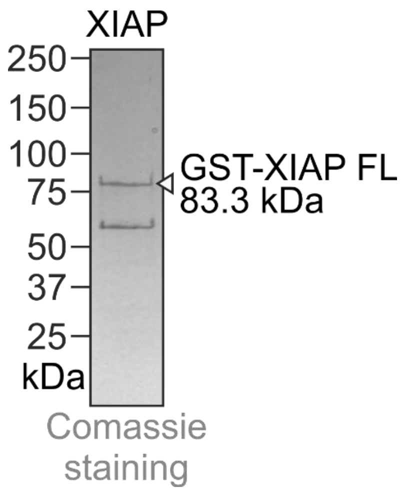

Appendix Figure S6 (related to Figure 3H,I and 4J). Purification of human full-length GST-XIAP.

Analysis of GST-tagged XIAP purification by Coomassie staining of SDS-PAGE.

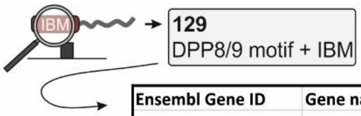

Ensembl Gene ID	Gene name	CCDS
ENSG00000163485	**ADORA1**	CCDS1434
ENSG00000004455	**AK2**	CCDS373
ENSG00000004455	**AK2**	CCDS374
ENSG00000004455	**AK2**	CCDS81294
ENSG00000004455	**AK2**	CCDS81295
ENSG00000004455	**AK2**	CCDS81296
ENSG00000002726	**AOC1**	CCDS43679
ENSG00000002726	**AOC1**	CCDS64797
ENSG00000105290	**APLP1**	CCDS32997
ENSG00000105290	**APLP1**	CCDS92600
ENSG00000132254	**ARFIP2**	CCDS55739
ENSG00000100325	**ASCC2**	CCDS13869
ENSG00000100325	**ASCC2**	CCDS56226
ENSG00000173918	**C1QTNF1**	CCDS11762
ENSG00000205129	**C4orf47**	CCDS47169
ENSG00000129993	**CBFA2T3**	CCDS10972
ENSG00000065883	**CDK13**	CCDS5461
ENSG00000065883	**CDK13**	CCDS5462
ENSG00000273777	**CEACAM20**	CCDS74390
ENSG00000273777	**CEACAM20**	CCDS74391
ENSG00000273777	**CEACAM20**	CCDS74392
ENSG00000273777	**CEACAM20**	CCDS74393
ENSG00000090539	**CHRD**	CCDS3266
ENSG00000090539	**CHRD**	CCDS77868
ENSG00000154080	**CHST9**	CCDS42422
ENSG00000154080	**CHST9**	CCDS58618
ENSG00000176435	**CLEC14A**	CCDS9667
ENSG00000114646	**CSPG5**	CCDS56252
ENSG00000155368	**DBI**	CCDS42741
ENSG00000188542	**DUSP28**	CCDS33418
ENSG00000144895	**EIF2A**	CCDS46935
ENSG00000144895	**EIF2A**	CCDS82859
ENSG00000144895	**EIF2A**	CCDS82860
ENSG00000144895	**EIF2A**	CCDS82861
ENSG00000178568	**ERBB4**	CCDS2394
ENSG00000178568	**ERBB4**	CCDS42811
ENSG00000178607	**ERN1**	CCDS45762
ENSG00000158869	**FCER1G**	CCDS1225
ENSG00000089327	**FXYD5**	CCDS12447
ENSG00000089327	**FXYD5**	CCDS82328
ENSG00000111087	**GLI1**	CCDS53807
ENSG00000125772	**GPCPD1**	CCDS13090
ENSG00000133937	**GSC**	CCDS9930
ENSG00000128731	**HERC2**	CCDS10021
ENSG00000197921	**HES5**	CCDS41233
ENSG00000160223	**ICOSLG**	CCDS93102
ENSG00000168811	**IL12A**	CCDS93420
ENSG00000068745	**IP6K2**	CCDS2777
ENSG00000068745	**IP6K2**	CCDS33752
ENSG00000068745	**IP6K2**	CCDS54579
ENSG00000132321	**IQCA1**	CCDS74677
ENSG00000161999	**JMJD8**	CCDS45369
ENSG00000161999	**JMJD8**	CCDS92074
ENSG00000161999	**JMJD8**	CCDS92075
ENSG00000144445	**KANSL1L**	CCDS33370
ENSG00000144445	**KANSL1L**	CCDS77519
ENSG00000135519	**KCNH3**	CCDS8786
ENSG00000087299	**L2HGDH**	CCDS9698
ENSG00000143815	**LBR**	CCDS1545
ENSG00000140506	**LMAN1L**	CCDS10270
ENSG00000254726	**MEX3A**	CCDS53377
ENSG00000178053	**MLF1**	CCDS93416
ENSG00000103152	**MPG**	CCDS32345
ENSG00000101181	**MTG2**	CCDS13492

Ensembl Gene ID	Gene name	CCDS
ENSG00000114026	**OGG1**	CCDS2576
ENSG00000114026	**OGG1**	CCDS2577
ENSG00000114026	**OGG1**	CCDS2578
ENSG00000114026	**OGG1**	CCDS2579
ENSG00000114026	**OGG1**	CCDS2580
ENSG00000114026	**OGG1**	CCDS2581
ENSG00000114026	**OGG1**	CCDS43046
ENSG00000114026	**OGG1**	CCDS46742
ENSG00000116774	**OLFML3**	CCDS870
ENSG00000076641	**PAG1**	CCDS6227
ENSG00000188677	**PARVB**	CCDS74874
ENSG00000056487	**PHF21B**	CCDS63504
ENSG00000130827	**PLXNA3**	CCDS14752
ENSG00000009830	**POMT2**	CCDS9857
ENSG00000189099	**PRSS48**	CCDS47145
ENSG00000103479	**RBL2**	CCDS10748
ENSG00000089902	**RCOR1**	CCDS9974
ENSG00000167771	**RCOR2**	CCDS8052
ENSG00000163515	**RETNLB**	CCDS2953
ENSG00000165799	**RNASE7**	CCDS41914
ENSG00000173431	**RNASE8**	CCDS9567
ENSG00000212864	**RNF208**	CCDS7037
ENSG00000197747	**S100A10**	CCDS1008
ENSG00000135622	**SEMA4F**	CCDS1955
ENSG00000135622	**SEMA4F**	CCDS62942
ENSG00000135622	**SEMA4F**	CCDS74529
ENSG00000099995	**SF3A1**	CCDS13875
ENSG00000113558	**SKP1**	CCDS4171
ENSG00000113558	**SKP1**	CCDS4172
ENSG00000155380	**SLC16A1**	CCDS858
ENSG00000173638	**SLC19A1**	CCDS13725
ENSG00000173638	**SLC19A1**	CCDS56218
ENSG00000205060	**SLC35B4**	CCDS34756
ENSG00000121871	**SLITRK3**	CCDS3197
ENSG00000132207	**SLX1A**	CCDS32431
ENSG00000132207	**SLX1A**	CCDS32432
ENSG00000181625	**SLX1B**	CCDS10648
ENSG00000181625	**SLX1B**	CCDS10649
ENSG00000198732	**SMOC1**	CCDS32110
ENSG00000198732	**SMOC1**	CCDS9798
ENSG00000152377	**SPOCK1**	CCDS4191
ENSG00000160075	**SSU72**	CCDS32
ENSG00000158246	**TENT5B**	CCDS294
ENSG00000129028	**THAP10**	CCDS10237
ENSG00000151952	**TMEM132D**	CCDS9266
ENSG00000125895	**TMEM74B**	CCDS13011
ENSG00000139921	**TMX1**	CCDS41953
ENSG00000284844	**TOMT**	CCDS91529
ENSG00000111669	**TPI1**	CCDS8566
ENSG00000239264	**TXNDC5**	CCDS4505
ENSG00000115446	**UNC50**	CCDS2035
ENSG00000104219	**ZDHHC2**	CCDS47810
ENSG00000156599	**ZDHHC5**	CCDS7965
ENSG00000166140	**ZFYVE19**	CCDS58353
ENSG00000164134	NAA15	CCDS43270
ENSG00000164134	NAA15	CCDS93635
ENSG00000117650	NEK2	CCDS1500
ENSG00000117650	NEK2	CCDS55682
ENSG00000117650	NEK2	CCDS73024
ENSG00000131196	NFATC1	CCDS12015
ENSG00000131196	NFATC1	CCDS59326
ENSG00000131196	NFATC1	CCDS62467
ENSG00000131196	NFATC1	CCDS62468
ENSG00000171246	NPTX1	CCDS32762
ENSG00000174145	NWD2	CCDS47040

Appendix Figure S7 (related to Figure 4A,B). DPP8 and DPP9 potentially unmask IBMs at the N-terminus of 129 cellular proteins.

A

STPLLTVR2$^+$ (PEP $1.32E^{-7}$) - DPP8/9 cleaved N-terminus in HEK WT
20240425_TB_SA_EIF2A_WT_20240428150220.raw (44.72 min)

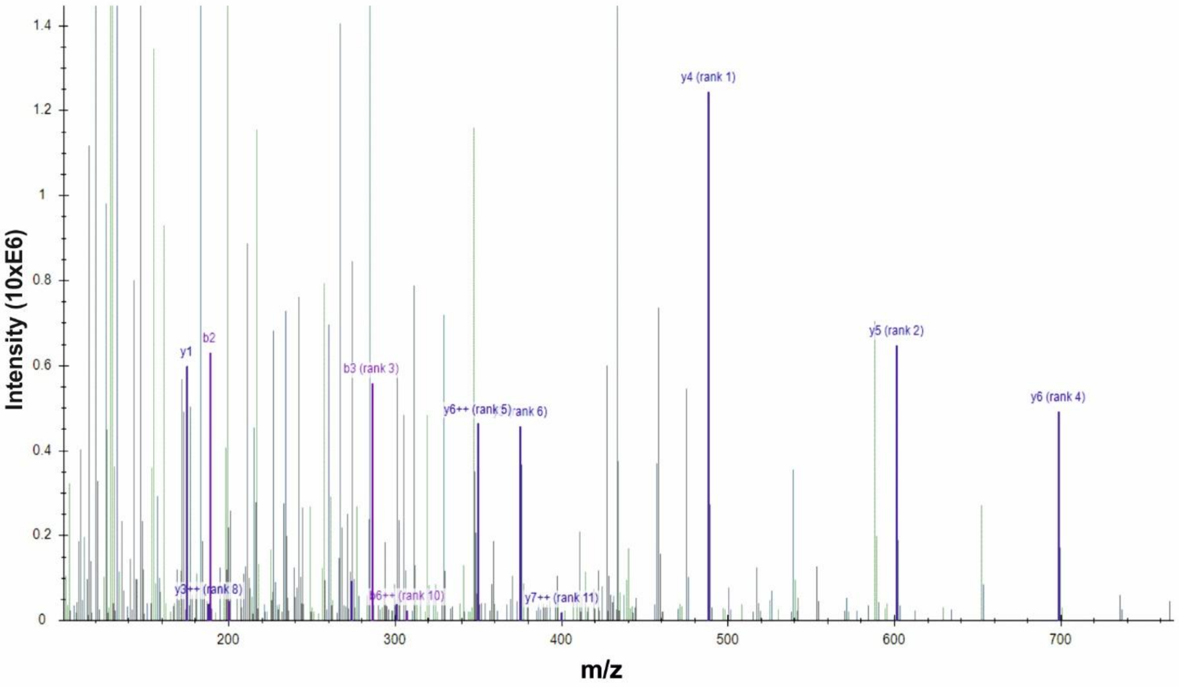

B

STPLLTVR2$^+$ (PEP $2.59E^{6}$) - DPP8/9 cleaved N-terminus in HEK DPP9 KO
2024_TB_SA_EIF2A_KO_20240428165944.raw (44.41 min)

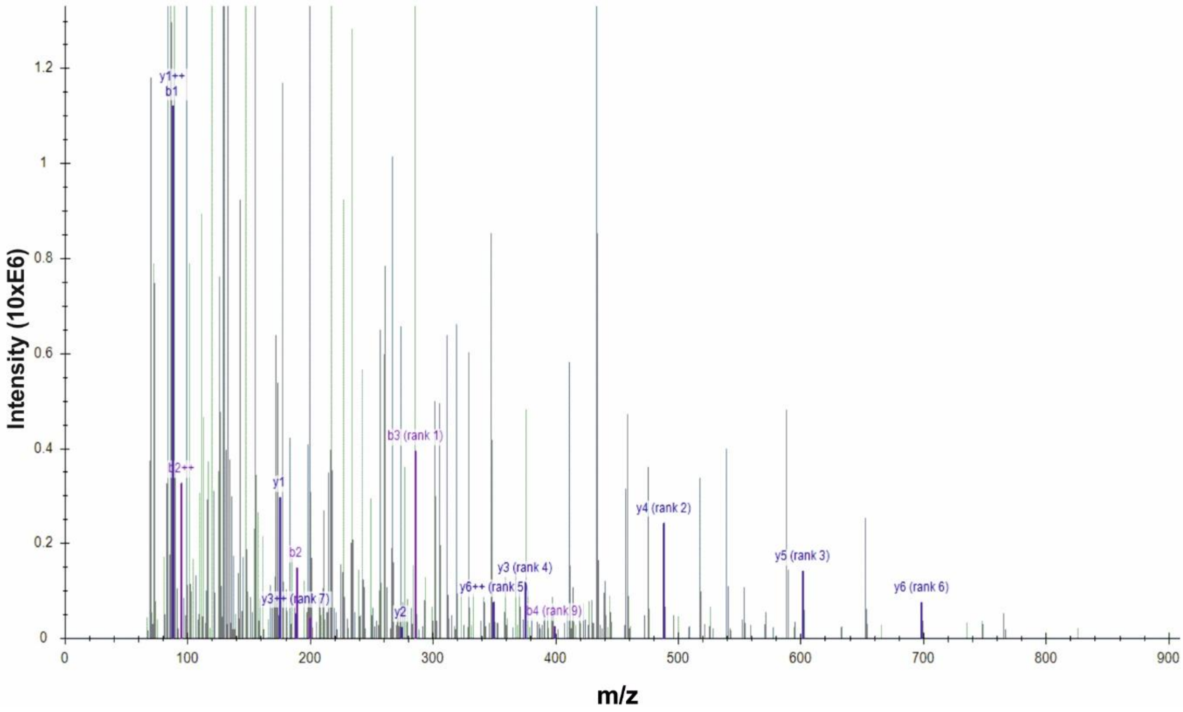

C

acSTPLLTVR2$^+$ (PEP 2.56E^{-6}) - acetylated DPP8/9 cleaved N-terminus in HEK WT
20240425_TB_SA_EIF2A_WT_20240428150220.raw (65.17 min)

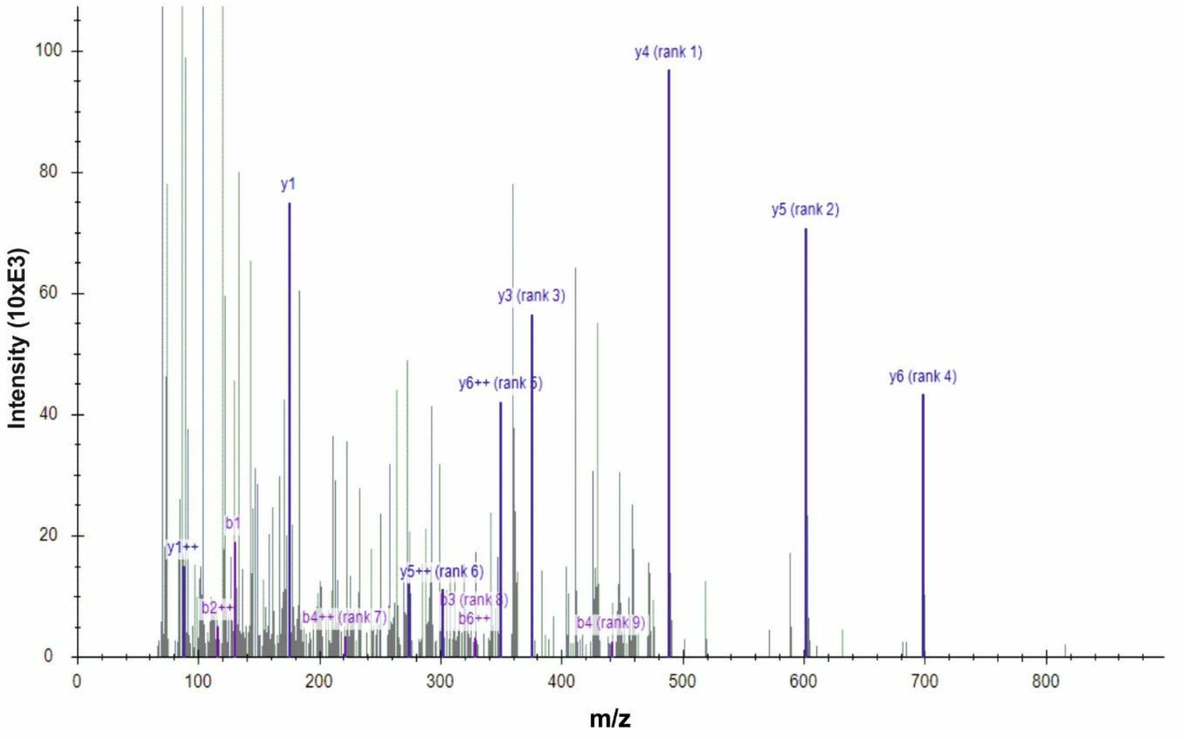

D

acSTPLLTVR2$^+$ (PEP 2.60E^{-6}) - acetylated DPP8/9 cleaved N-terminus in HEK DPP9 KO
20240425_TB_SA_EIF2A_KO_20240428165994.raw (64.82 min)

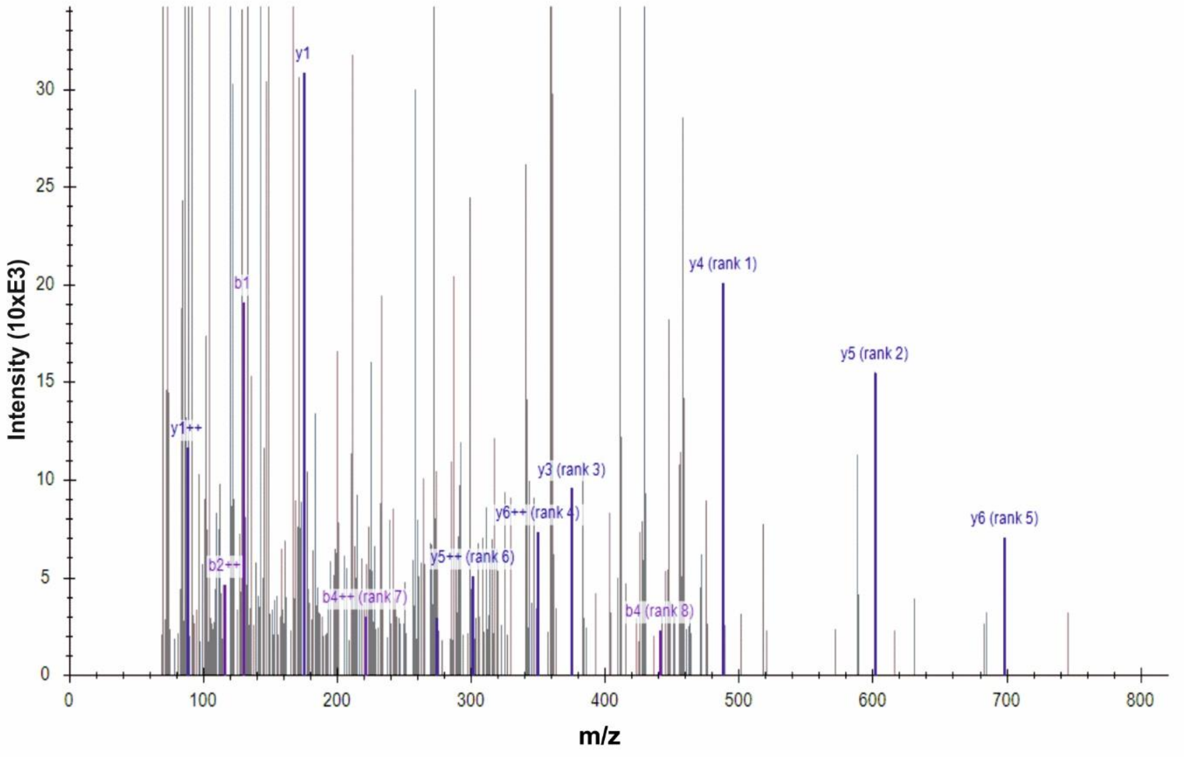

E

APSTPLLTVR2⁺ (PEP 0.09)- MetAP cleaved N-terminus in HEK WT

20240425_TB_SA_EIF2A_WT_20240428150220.raw (50.97 min)

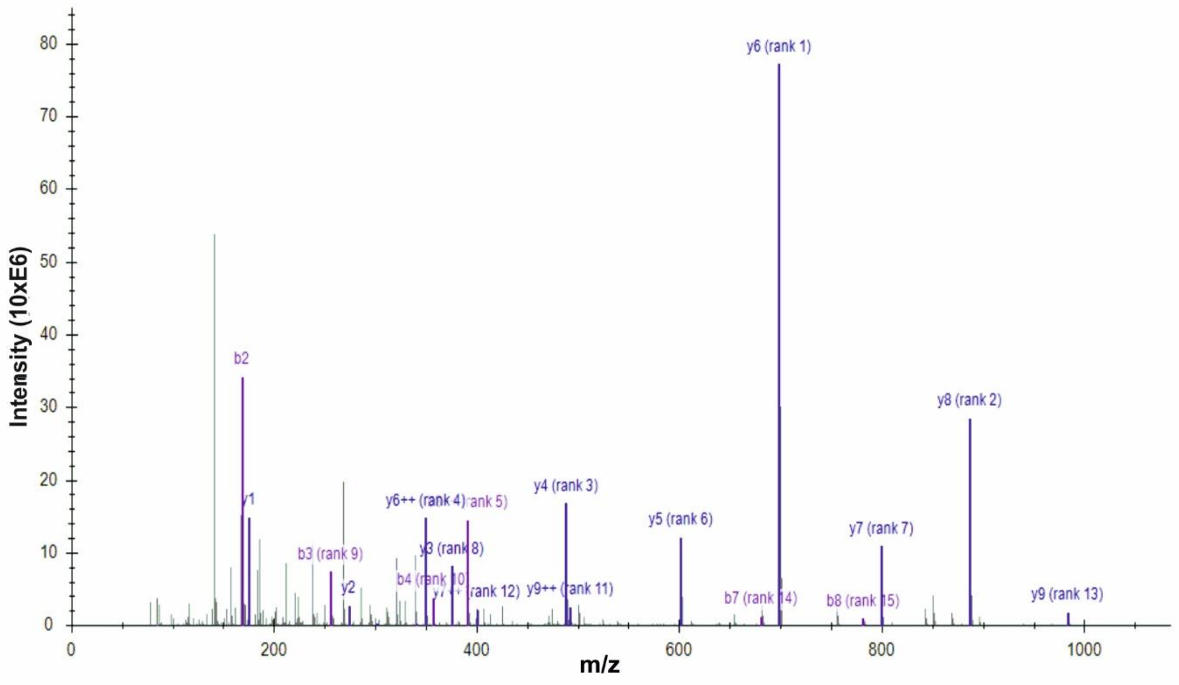

F

APSTPLLTVR2⁺ (PEP 0.09) - MetAP cleaved N-terminus in HEK DPP9 KO

20240425_TB_SA_EIF2A_KO_20240428165944.raw (50.61 min)

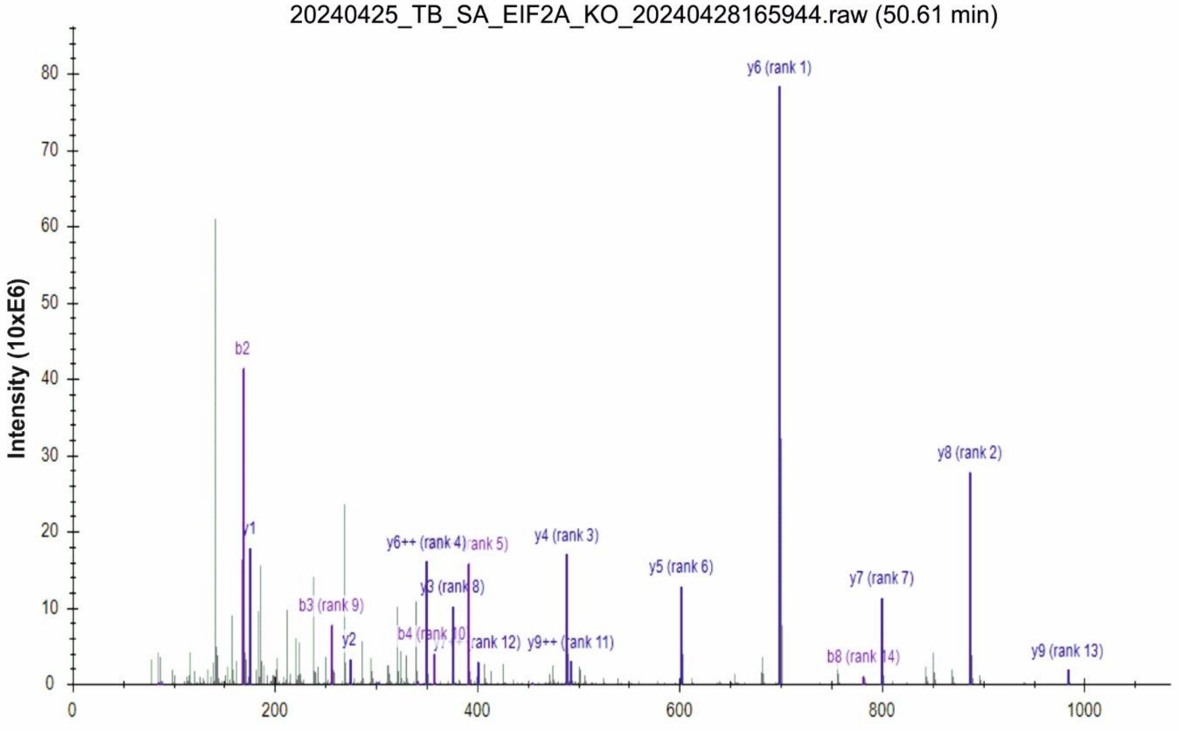

G

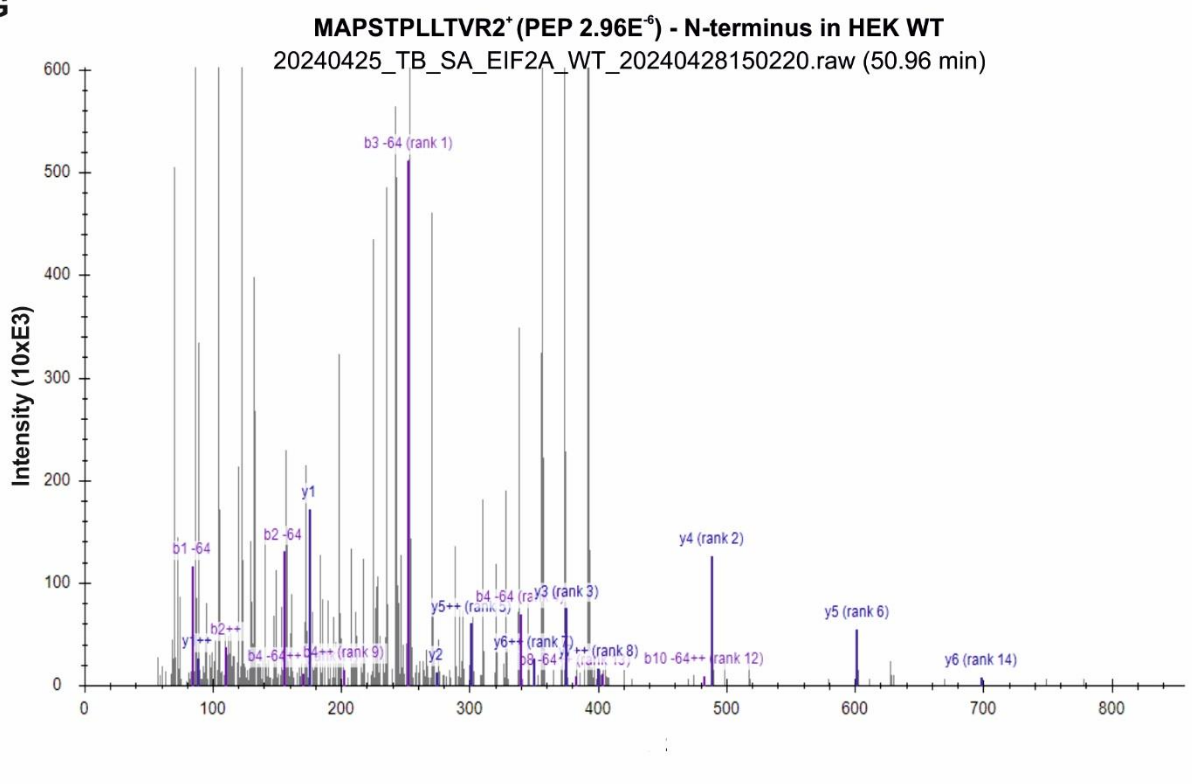
MAPSTPLLTVR2+ (PEP 2.96E-6) - N-terminus in HEK WT
20240425_TB_SA_EIF2A_WT_20240428150220.raw (50.96 min)
Intensity (10xE3)
b3 -64 (rank 1)
y1
b2 -64
b1 -64
y4 (rank 2)
y3 (rank 3)
y5 (rank 6)
y6 (rank 14)
y5++ (rank 5)
y6++ (rank 7)
b4 -64++ (rank 9)
b10 -64++ (rank 12)
b2++
y2

H

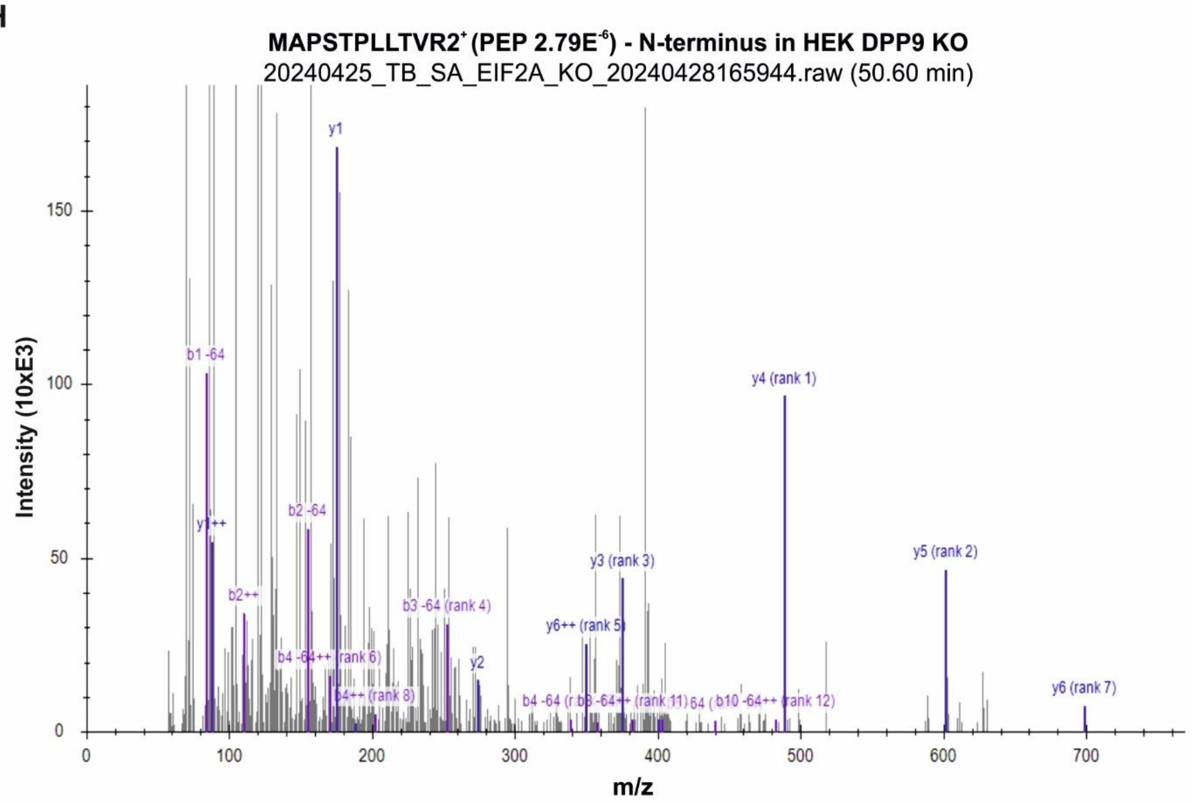
MAPSTPLLTVR2+ (PEP 2.79E-6) - N-terminus in HEK DPP9 KO
20240425_TB_SA_EIF2A_KO_20240428165944.raw (50.60 min)
Intensity (10xE3)
m/z
y1
b1 -64
y4 (rank 1)
b2 -64
y3 (rank 3)
y5 (rank 2)
b2++
b3 -64 (rank 4)
y6++ (rank 5)
b4 -64++ (rank 6)
b4++ (rank 8)
y2
b10 -64++ (rank 12)
y6 (rank 7)

Appendix Figure S8 (related to Figure 4C). MS2 spectra for EIF2A peptides identified in whole cellular lysates of HEK293 WT and DPP9 KO cells.

A,B. EIF2A N-terminal peptide NH_2-STPLLTV<u>R</u> in WT (A) and DPP9 KO (B) lysate.

C,D. EIF2A N-terminal peptide ac-NH_2-STPLLTV<u>R</u> in WT (C) and DPP9 KO (D) lysate.

E,F. EIF2A N-terminal peptide NH_2-APSTPLLTV<u>R</u> in WT (E) and DPP9 KO (F) lysates.

G,H. EIF2A N-terminal peptide NH_2-MAPSTPLLTV<u>R</u> in WT (G) and DPP9 KO (H) lysates.

APPENDIX REFERENCES

Bolgi O, Silva-Garcia M, Ross B, Pilla E, Kari V, Killisch M, Spitzner M, Stark N, Lenz C, Weiss K *et al* (2022) Dipeptidyl peptidase 9 triggers BRCA2 degradation and promotes DNA damage repair. *EMBO reports* 23: e54136-e54136

Finger Y, Habich M, Gerlich S, Urbanczyk S, van de Logt E, Koch J, Schu L, Lapacz KJ, Ali M, Petrungaro C *et al* (2020) Proteasomal degradation induced by DPP9-mediated processing competes with mitochondrial protein import. *The EMBO Journal* 39

Jumper J, Evans R, Pritzel A, Green T, Figurnov M, Ronneberger O, Tunyasuvunakool K, Bates R, Zidek A, Potapenko A *et al* (2021) Highly accurate protein structure prediction with AlphaFold. *Nature* 596: 583-589

Ran FA, Hsu PD, Wright J, Agarwala V, Scott DA, Zhang F (2013) Genome engineering using the CRISPR-Cas9 system. *Nat Protoc* 8: 2281-2308
