# Supplementary figures and images for: DPP8/9 processing of human AK2 unmasks an IAP binding motif
Source: EMBO Rep. 2025 May 1;26(11):2819–35. doi: 10.1038/s44319-025-00455-z (PMC12152192; doi:10.1038/s44319-025-00455-z)

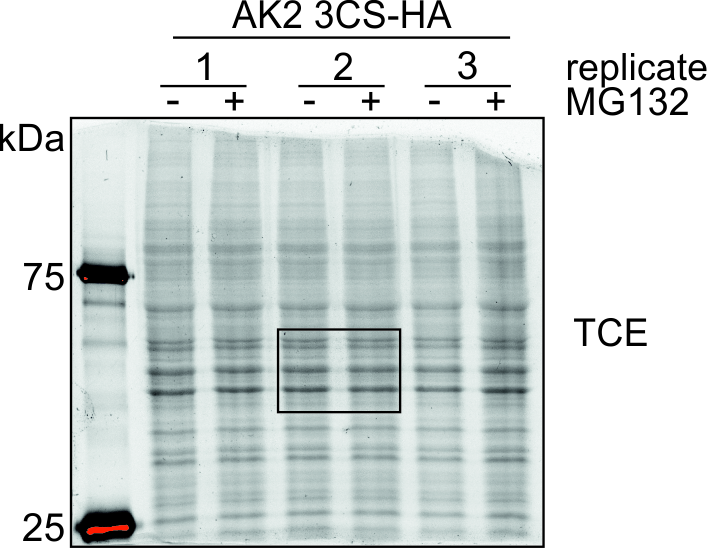

Supplement: Supplementary file 3 — Source data Fig. 1 [file 44319_2025_455_MOESM3_ESM.zip › Figure 1/1B/SDS-PAGE TCE.tif]

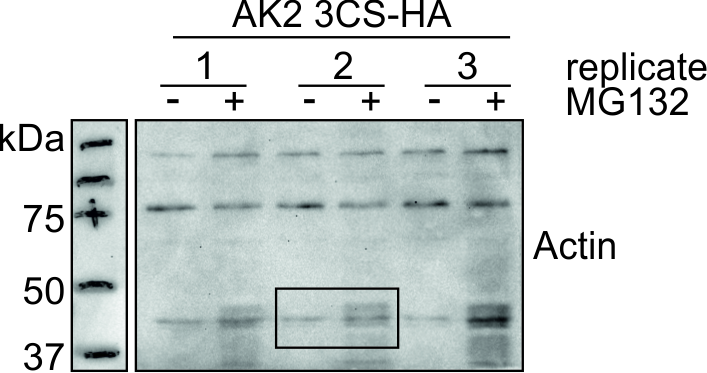

Supplement: Supplementary file 3 — Source data Fig. 1 [file 44319_2025_455_MOESM3_ESM.zip › Figure 1/1B/western Actin.tif]

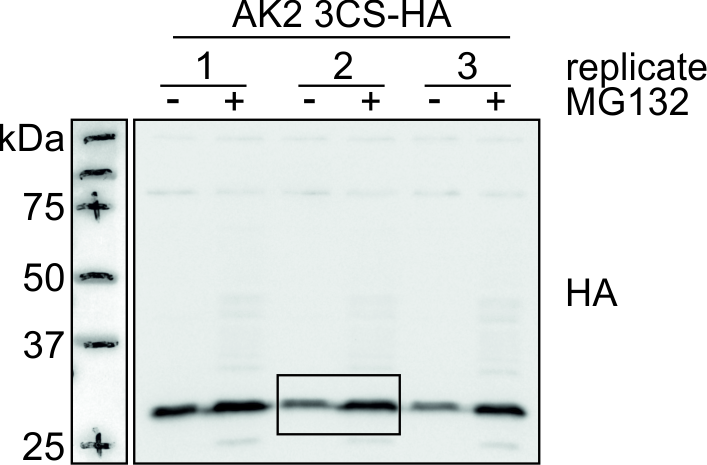

Supplement: Supplementary file 3 — Source data Fig. 1 [file 44319_2025_455_MOESM3_ESM.zip › Figure 1/1B/western HA.tif]

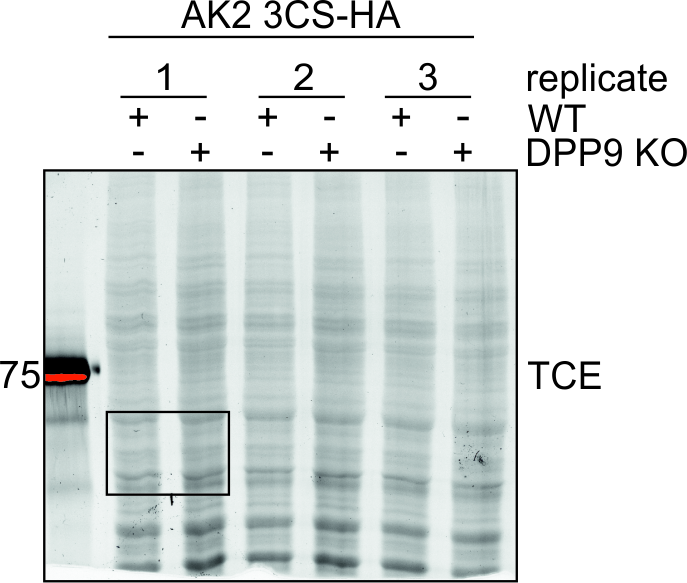

Supplement: Supplementary file 3 — Source data Fig. 1 [file 44319_2025_455_MOESM3_ESM.zip › Figure 1/1C/SDS-PAGE TCE.tif]

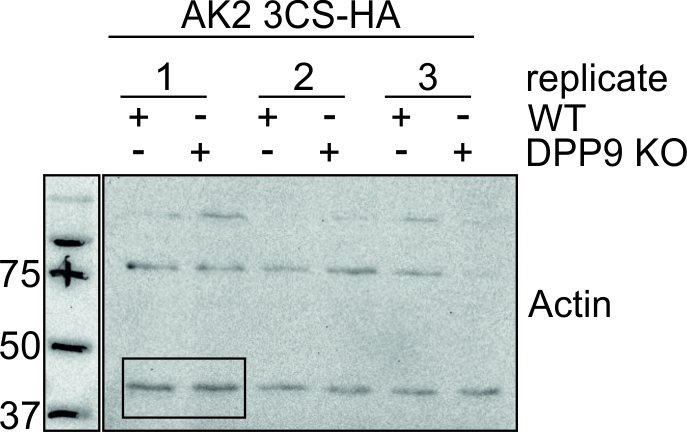

Supplement: Supplementary file 3 — Source data Fig. 1 [file 44319_2025_455_MOESM3_ESM.zip › Figure 1/1C/western Actin.tif]

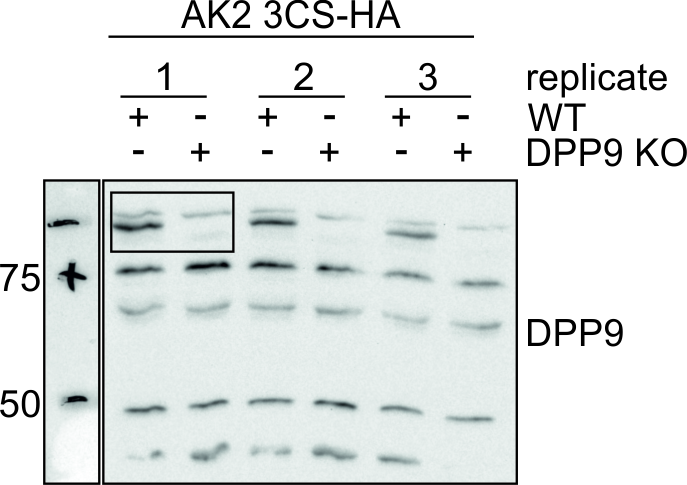

Supplement: Supplementary file 3 — Source data Fig. 1 [file 44319_2025_455_MOESM3_ESM.zip › Figure 1/1C/western DPP9.tif]

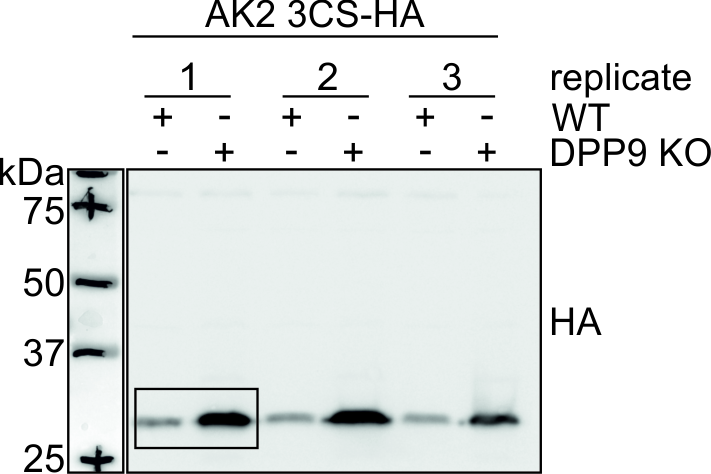

Supplement: Supplementary file 3 — Source data Fig. 1 [file 44319_2025_455_MOESM3_ESM.zip › Figure 1/1C/western HA.tif]

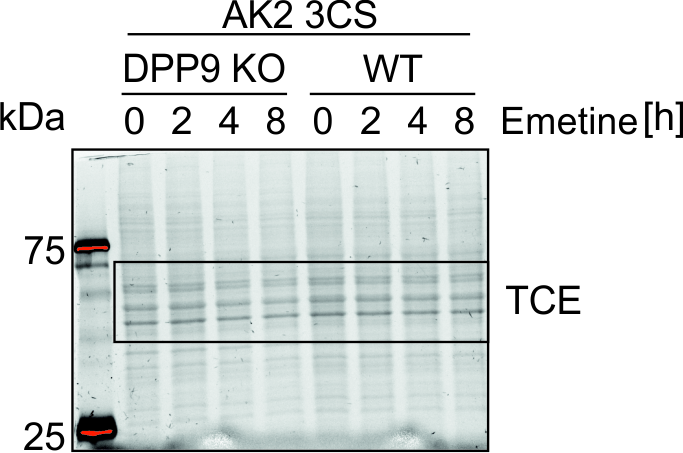

Supplement: Supplementary file 3 — Source data Fig. 1 [file 44319_2025_455_MOESM3_ESM.zip › Figure 1/1D/SDS-PAGE TCE.tif]

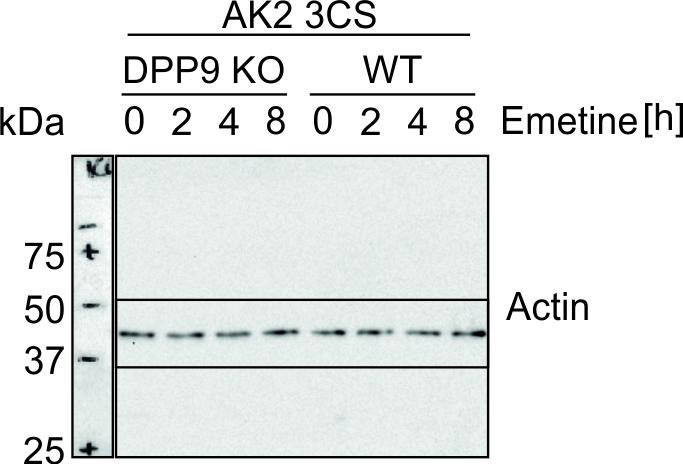

Supplement: Supplementary file 3 — Source data Fig. 1 [file 44319_2025_455_MOESM3_ESM.zip › Figure 1/1D/western Actin.tif]

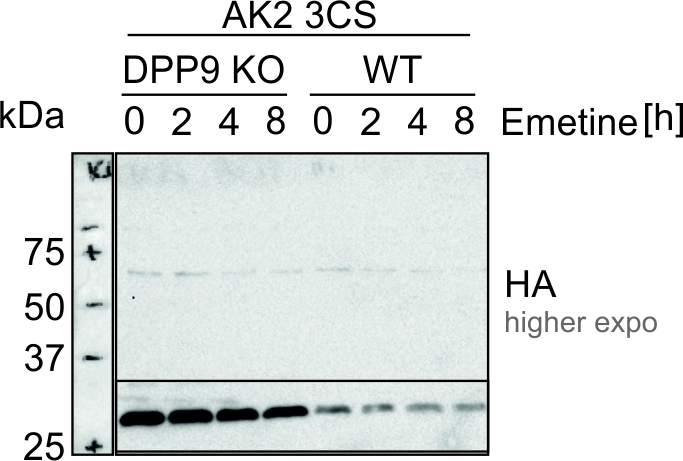

Supplement: Supplementary file 3 — Source data Fig. 1 [file 44319_2025_455_MOESM3_ESM.zip › Figure 1/1D/western HA higher expo.tif]

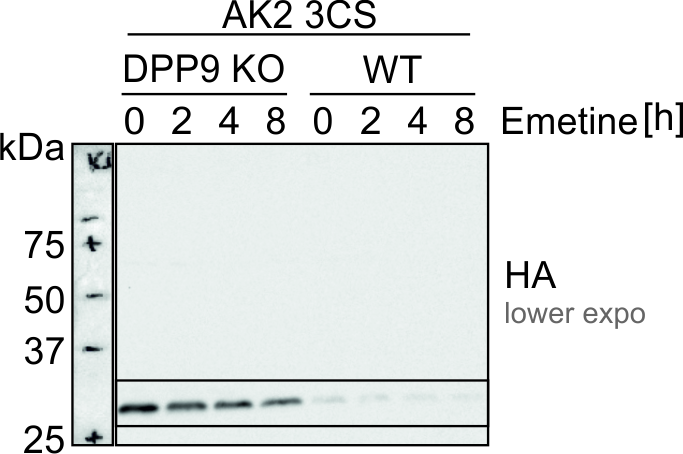

Supplement: Supplementary file 3 — Source data Fig. 1 [file 44319_2025_455_MOESM3_ESM.zip › Figure 1/1D/western HA lower expo.tif]

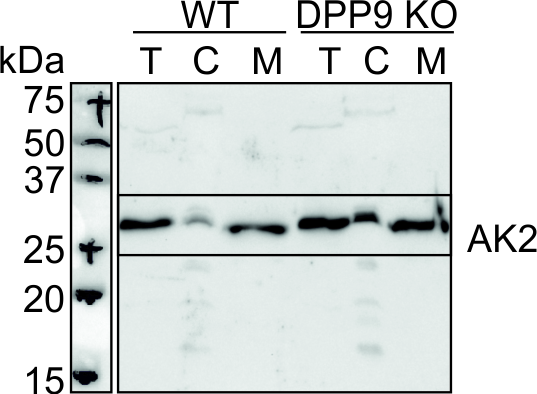

Supplement: Supplementary file 3 — Source data Fig. 1 [file 44319_2025_455_MOESM3_ESM.zip › Figure 1/1E/western AK2.tif]

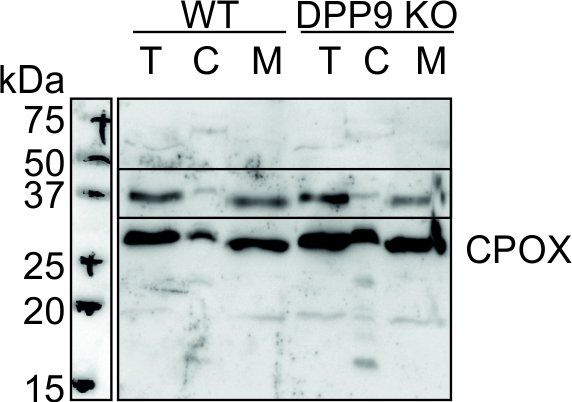

Supplement: Supplementary file 3 — Source data Fig. 1 [file 44319_2025_455_MOESM3_ESM.zip › Figure 1/1E/western CPOX.tif]

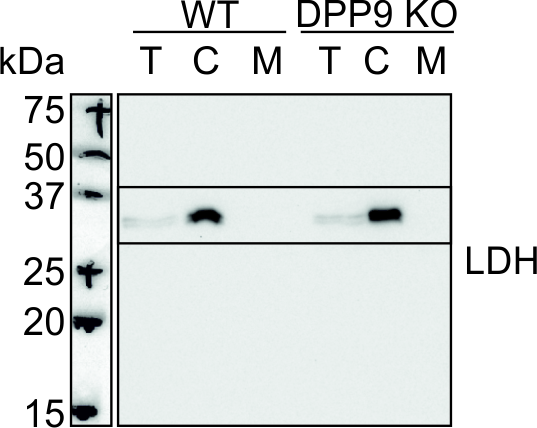

Supplement: Supplementary file 3 — Source data Fig. 1 [file 44319_2025_455_MOESM3_ESM.zip › Figure 1/1E/western LDH.tif]

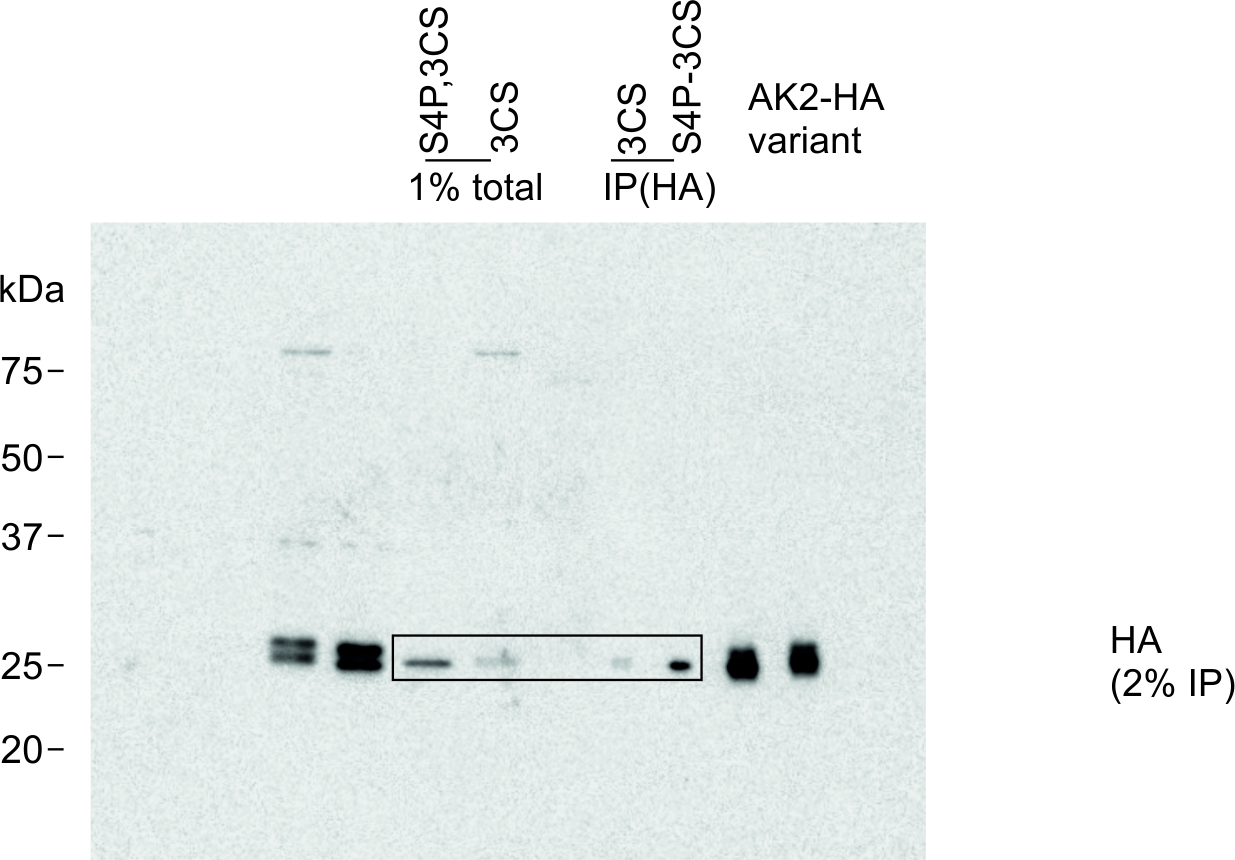

Supplement: Supplementary file 4 — Source data Fig. 2 [file 44319_2025_455_MOESM4_ESM.zip › Figure 2/2L/western HA.tif]

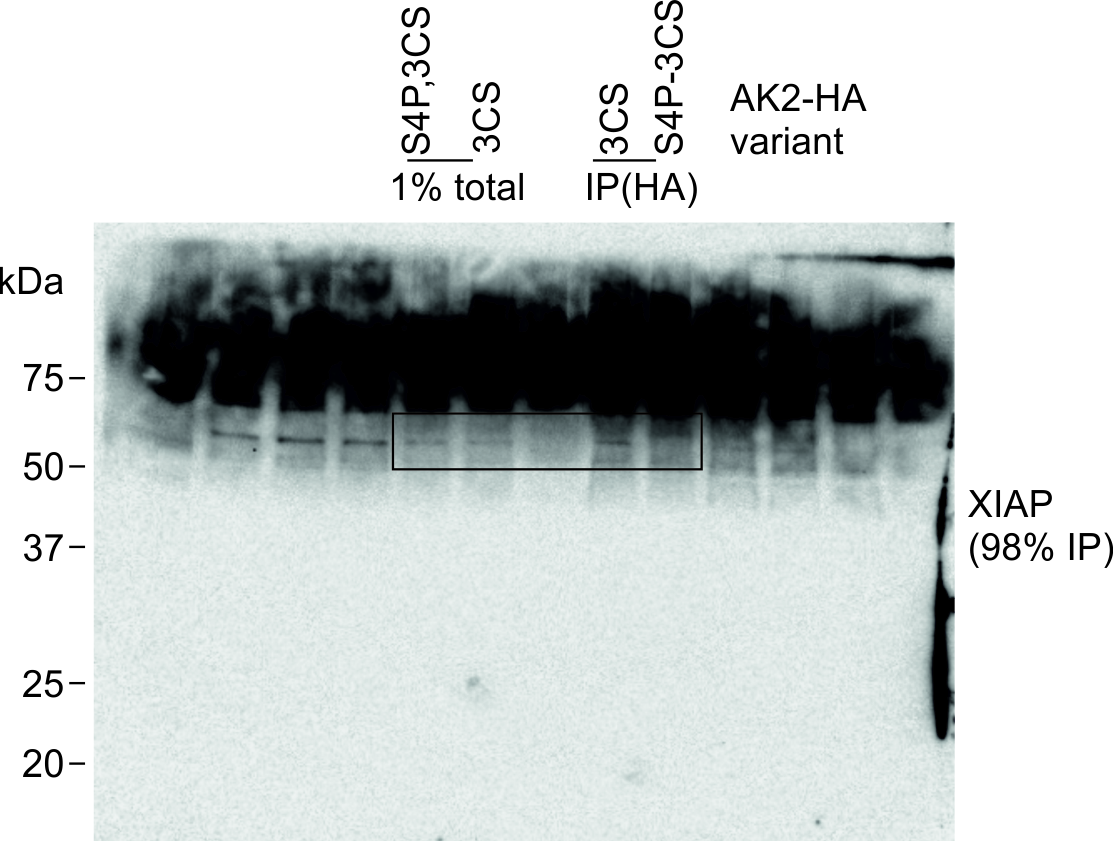

Supplement: Supplementary file 4 — Source data Fig. 2 [file 44319_2025_455_MOESM4_ESM.zip › Figure 2/2L/western XIAP.tif]

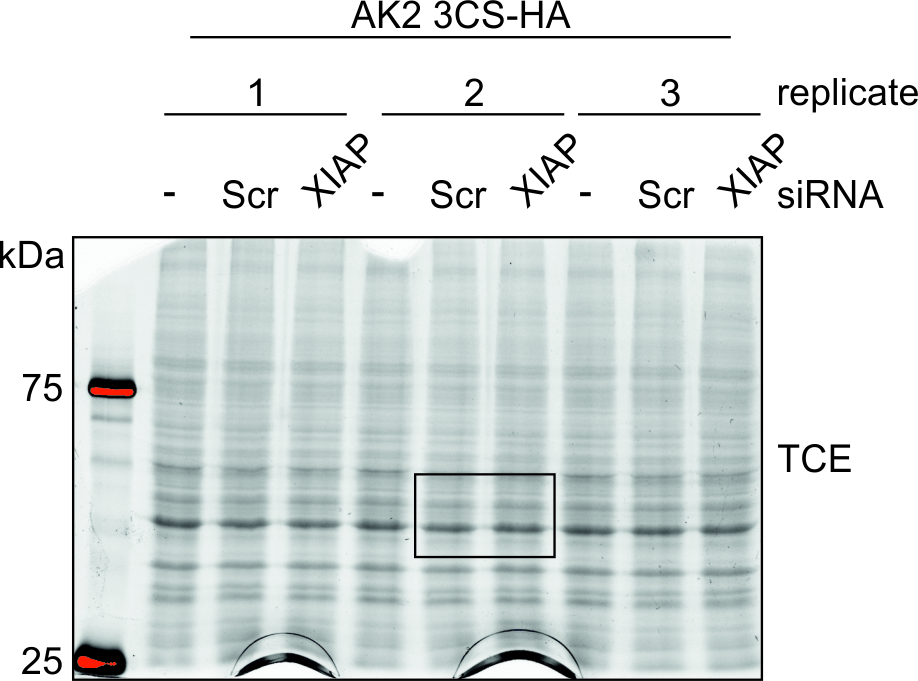

Supplement: Supplementary file 5 — Source data Fig. 3 [file 44319_2025_455_MOESM5_ESM.zip › Figure 3/3A/SDS-PAGE TCE for HA western.tif]

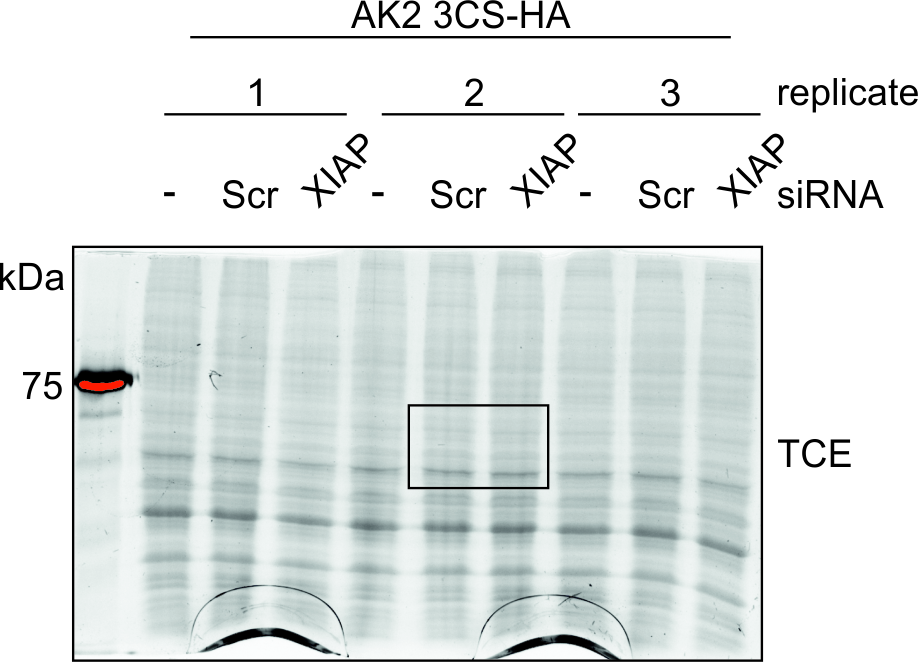

Supplement: Supplementary file 5 — Source data Fig. 3 [file 44319_2025_455_MOESM5_ESM.zip › Figure 3/3A/SDS-PAGE TCE for XIAP western.tif]

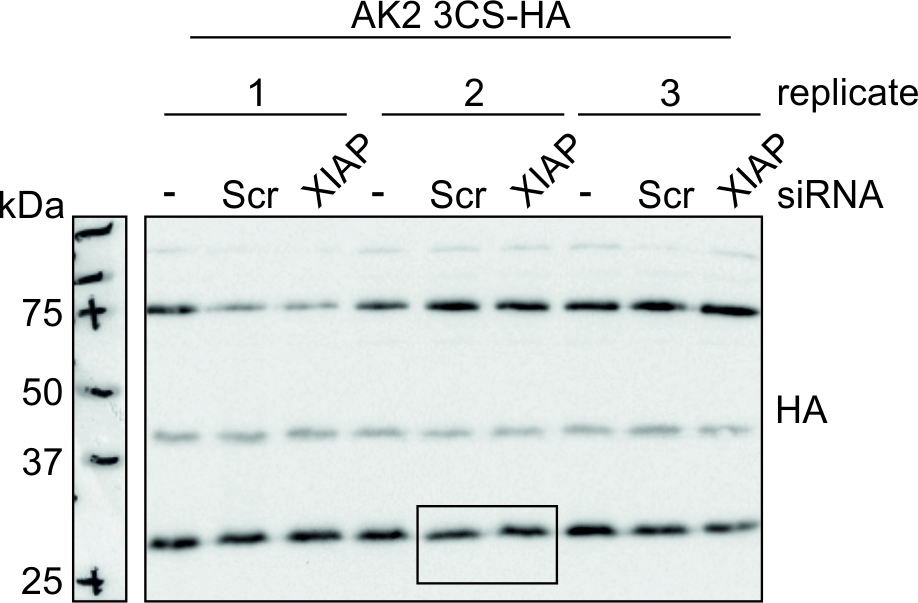

Supplement: Supplementary file 5 — Source data Fig. 3 [file 44319_2025_455_MOESM5_ESM.zip › Figure 3/3A/western HA.tif]

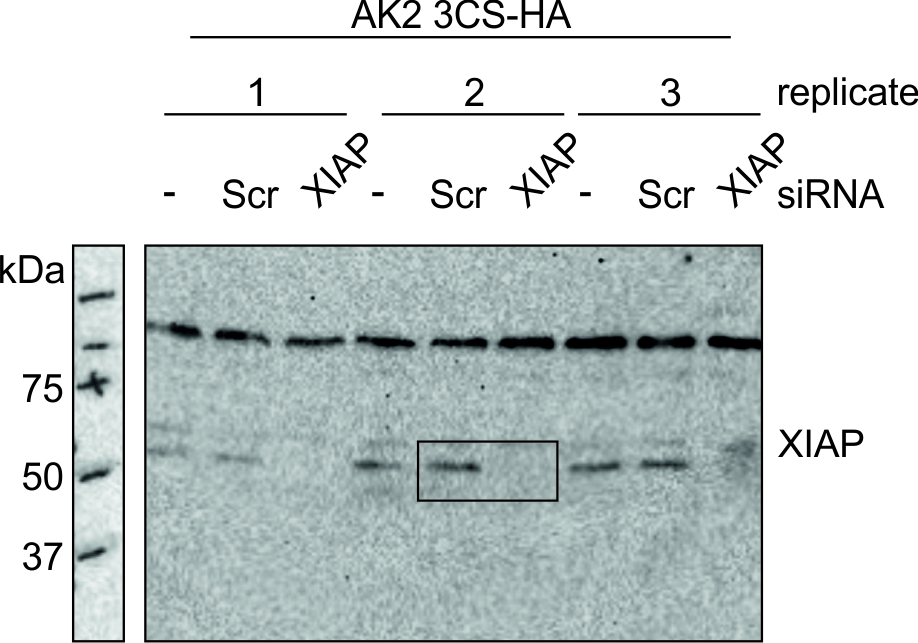

Supplement: Supplementary file 5 — Source data Fig. 3 [file 44319_2025_455_MOESM5_ESM.zip › Figure 3/3A/western XIAP.tif]

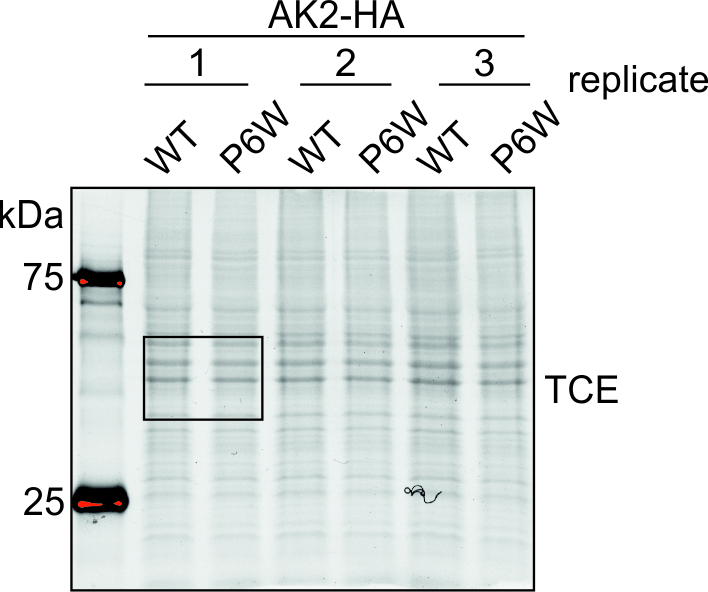

Supplement: Supplementary file 5 — Source data Fig. 3 [file 44319_2025_455_MOESM5_ESM.zip › Figure 3/3B/SDS-PAGE TCE.tif]

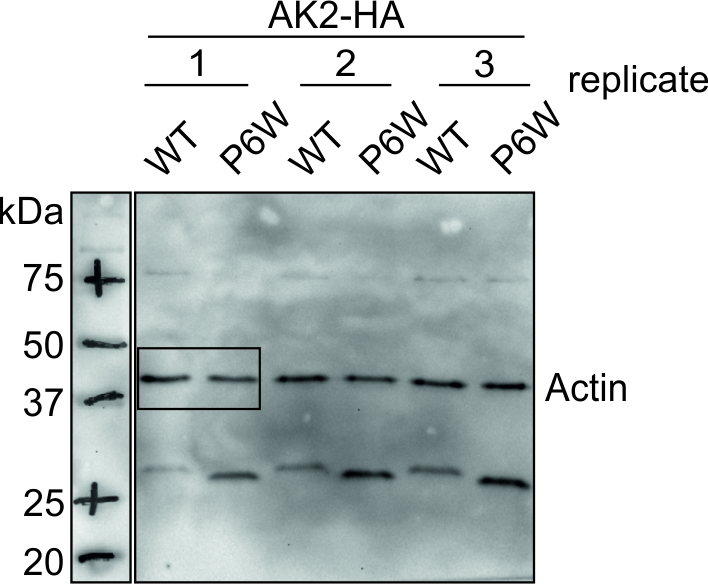

Supplement: Supplementary file 5 — Source data Fig. 3 [file 44319_2025_455_MOESM5_ESM.zip › Figure 3/3B/western Actin.tif]

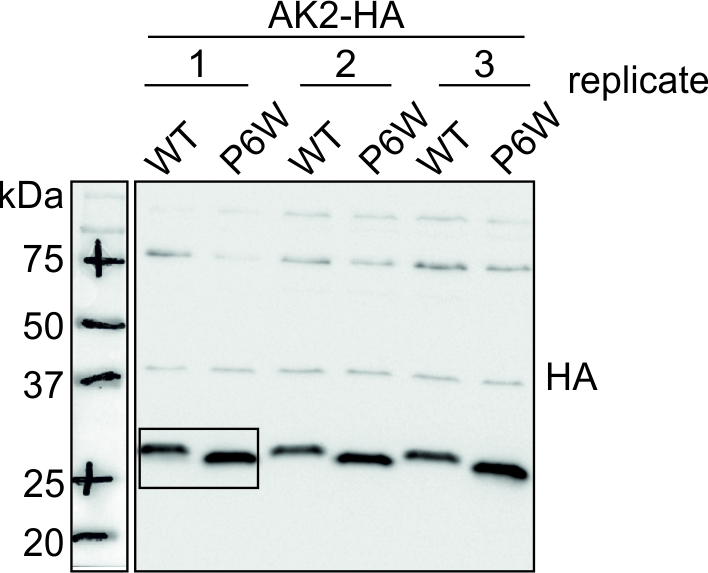

Supplement: Supplementary file 5 — Source data Fig. 3 [file 44319_2025_455_MOESM5_ESM.zip › Figure 3/3B/western HA.tif]

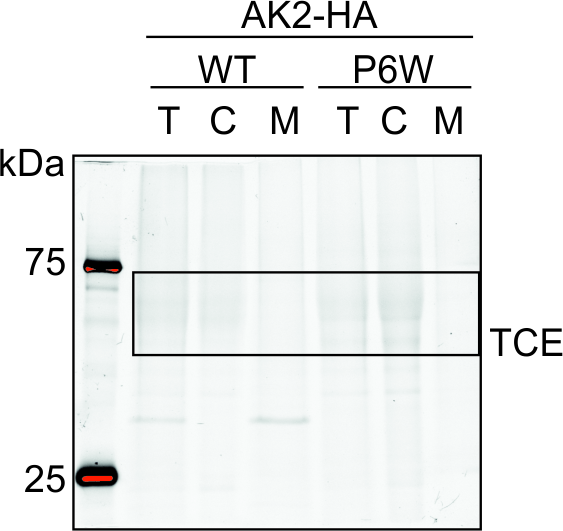

Supplement: Supplementary file 5 — Source data Fig. 3 [file 44319_2025_455_MOESM5_ESM.zip › Figure 3/3C/SDS-PAGE TCE.tif]

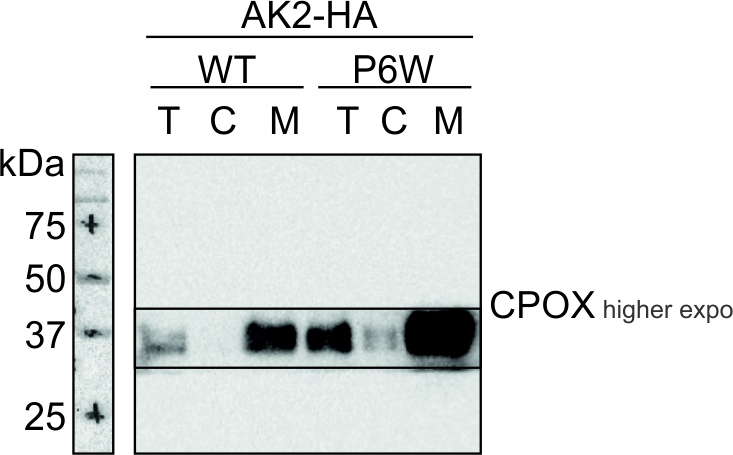

Supplement: Supplementary file 5 — Source data Fig. 3 [file 44319_2025_455_MOESM5_ESM.zip › Figure 3/3C/western CPOX higher expo.tif]

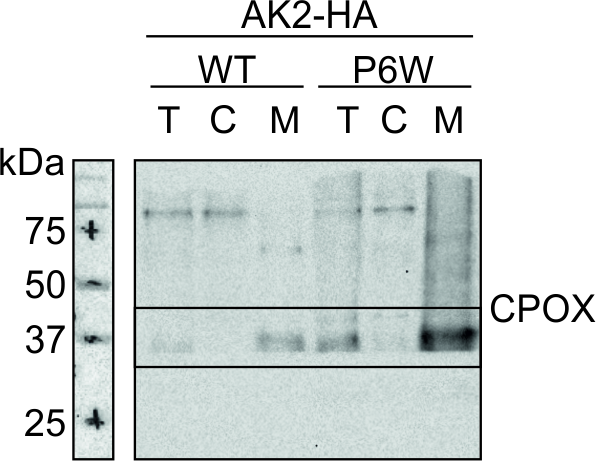

Supplement: Supplementary file 5 — Source data Fig. 3 [file 44319_2025_455_MOESM5_ESM.zip › Figure 3/3C/western CPOX.tif]

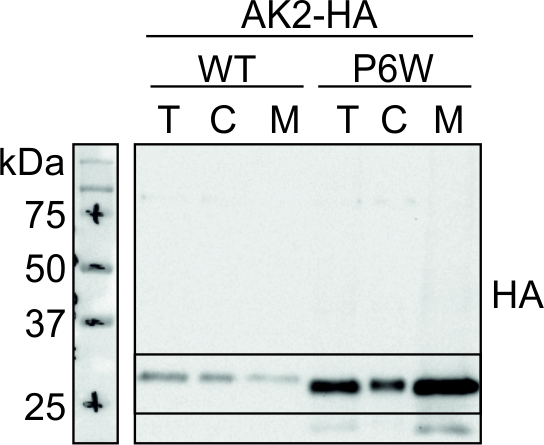

Supplement: Supplementary file 5 — Source data Fig. 3 [file 44319_2025_455_MOESM5_ESM.zip › Figure 3/3C/western HA.tif]

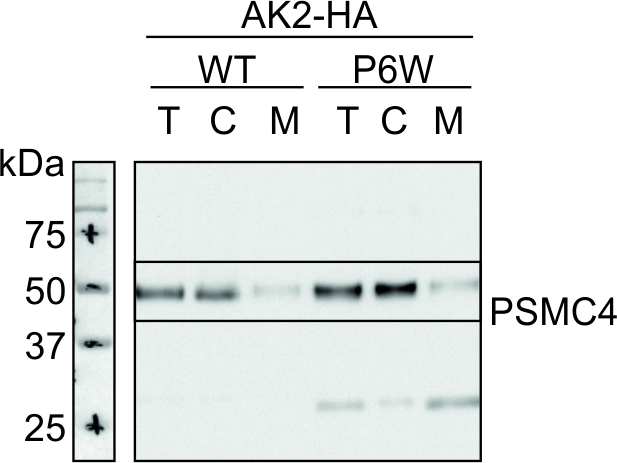

Supplement: Supplementary file 5 — Source data Fig. 3 [file 44319_2025_455_MOESM5_ESM.zip › Figure 3/3C/western PSMC4.tif]

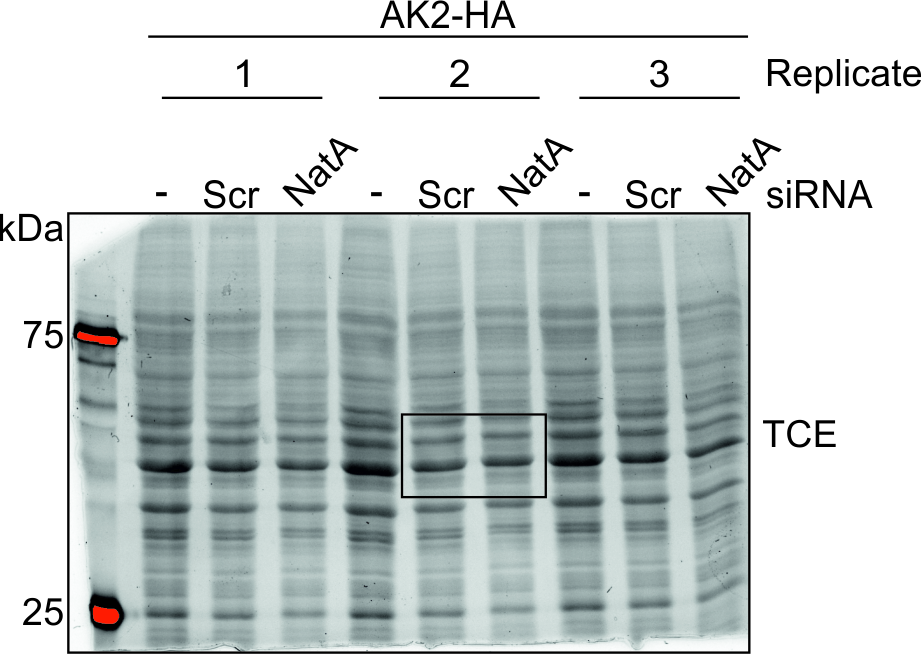

Supplement: Supplementary file 5 — Source data Fig. 3 [file 44319_2025_455_MOESM5_ESM.zip › Figure 3/3F/SDS-PAGE TCE.tif]

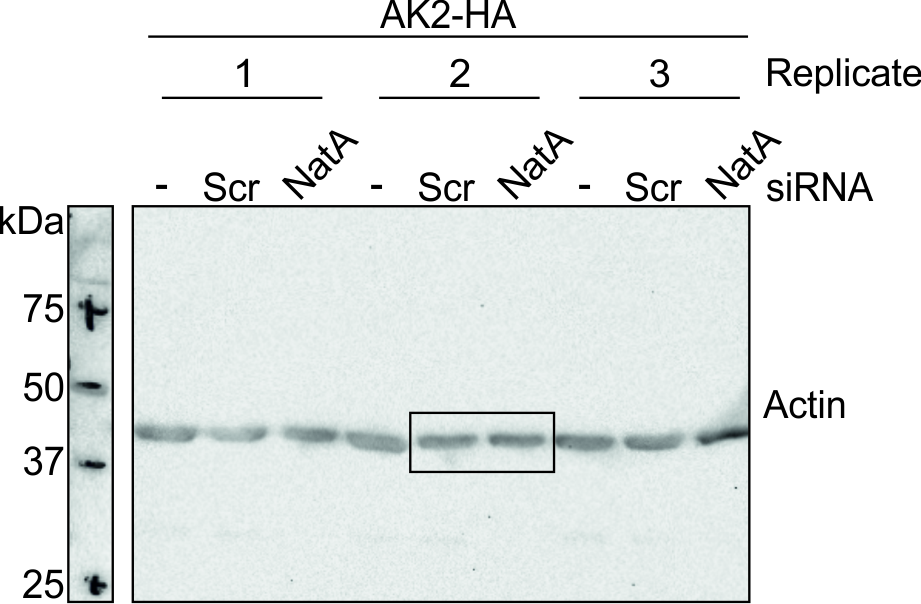

Supplement: Supplementary file 5 — Source data Fig. 3 [file 44319_2025_455_MOESM5_ESM.zip › Figure 3/3F/western actin 1.tif]

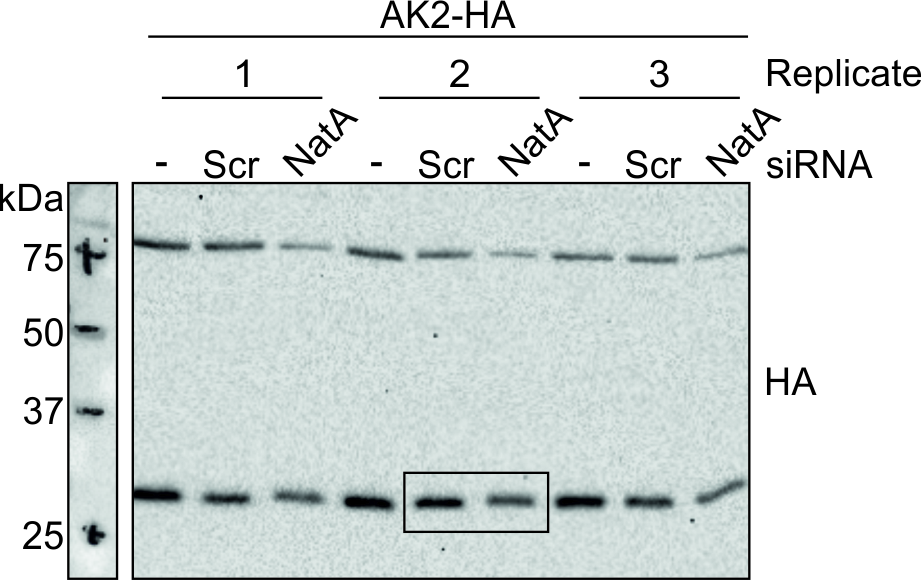

Supplement: Supplementary file 5 — Source data Fig. 3 [file 44319_2025_455_MOESM5_ESM.zip › Figure 3/3F/western HA.tif]

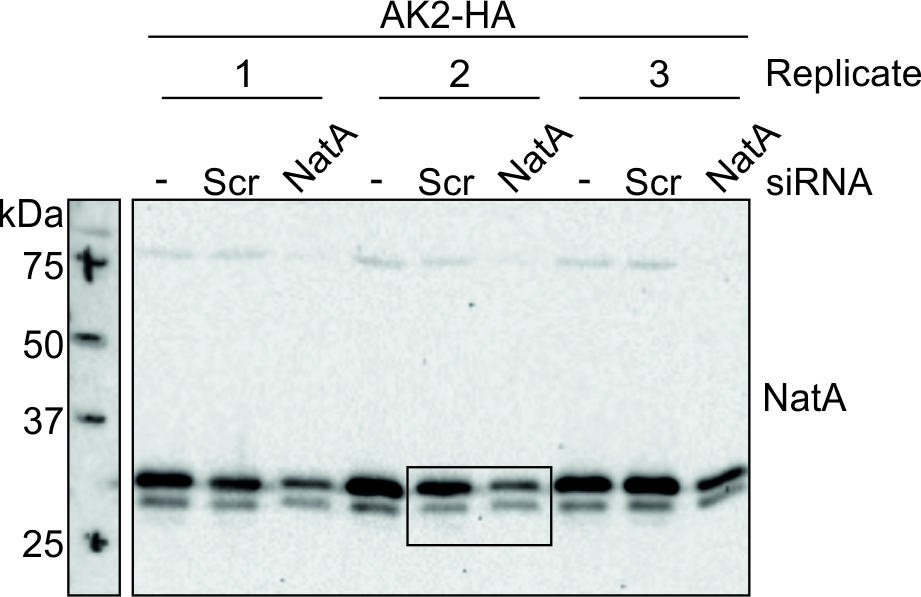

Supplement: Supplementary file 5 — Source data Fig. 3 [file 44319_2025_455_MOESM5_ESM.zip › Figure 3/3F/western NatA.tif]

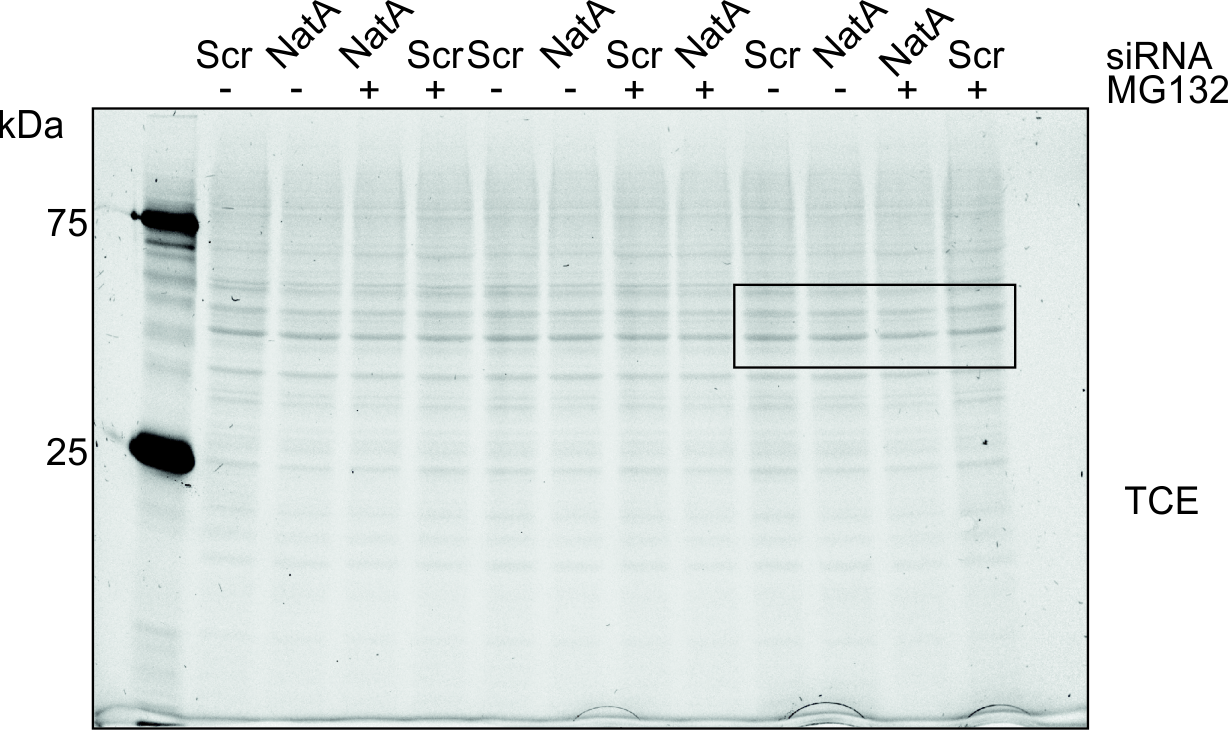

Supplement: Supplementary file 5 — Source data Fig. 3 [file 44319_2025_455_MOESM5_ESM.zip › Figure 3/3G/SDS-PAGE TCE.tif]

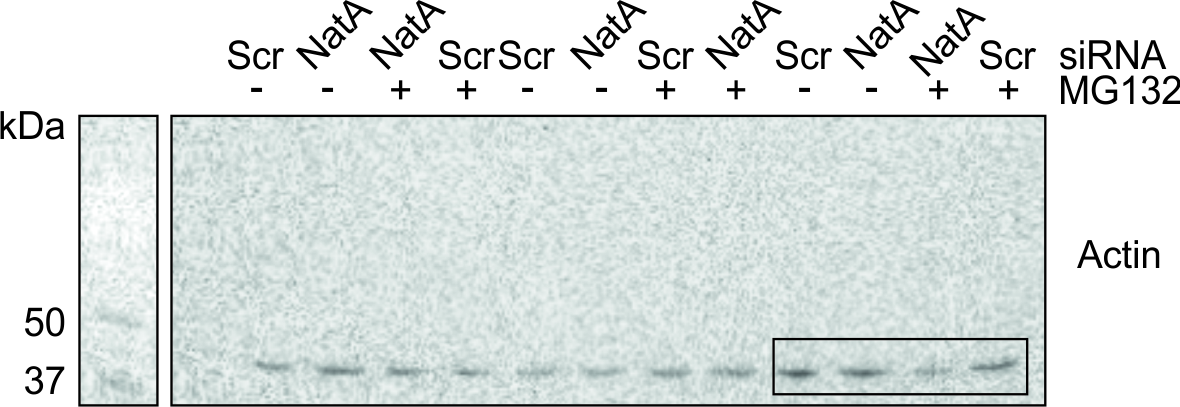

Supplement: Supplementary file 5 — Source data Fig. 3 [file 44319_2025_455_MOESM5_ESM.zip › Figure 3/3G/western Actin.tif]

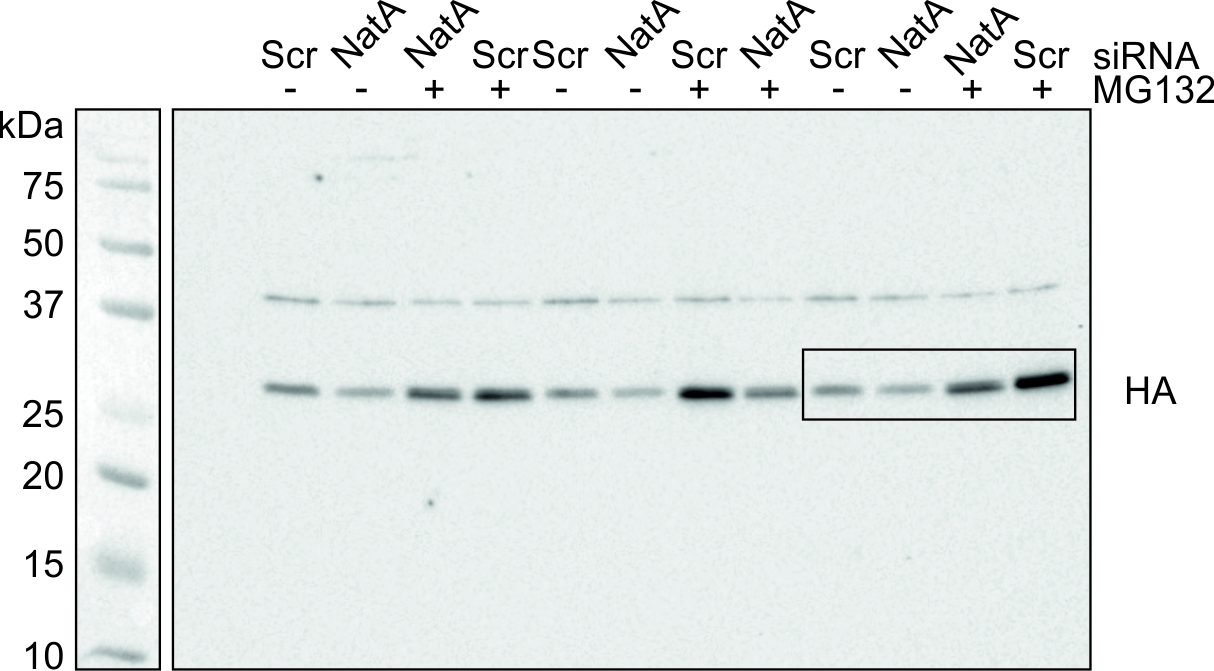

Supplement: Supplementary file 5 — Source data Fig. 3 [file 44319_2025_455_MOESM5_ESM.zip › Figure 3/3G/western HA.tif]

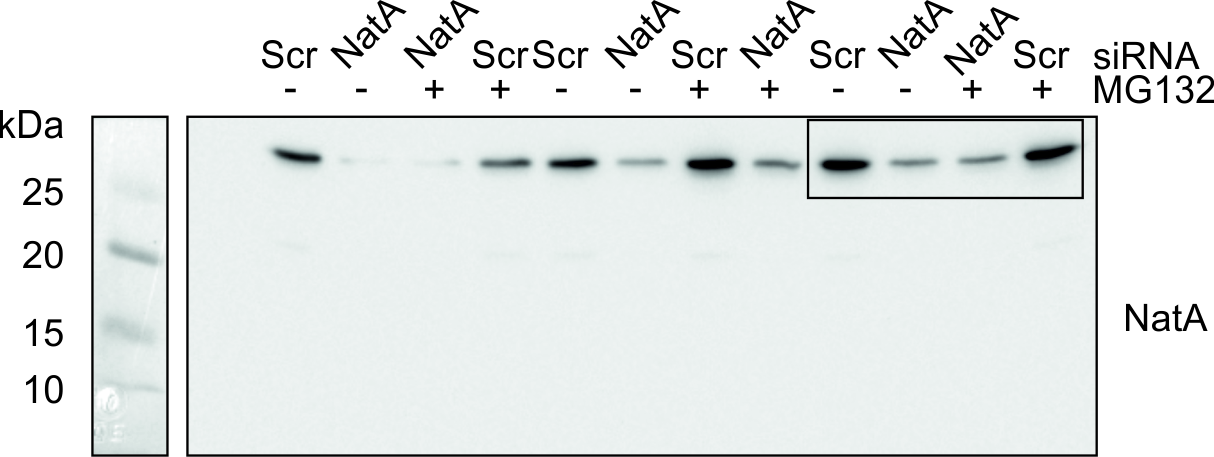

Supplement: Supplementary file 5 — Source data Fig. 3 [file 44319_2025_455_MOESM5_ESM.zip › Figure 3/3G/western NatA.tif]

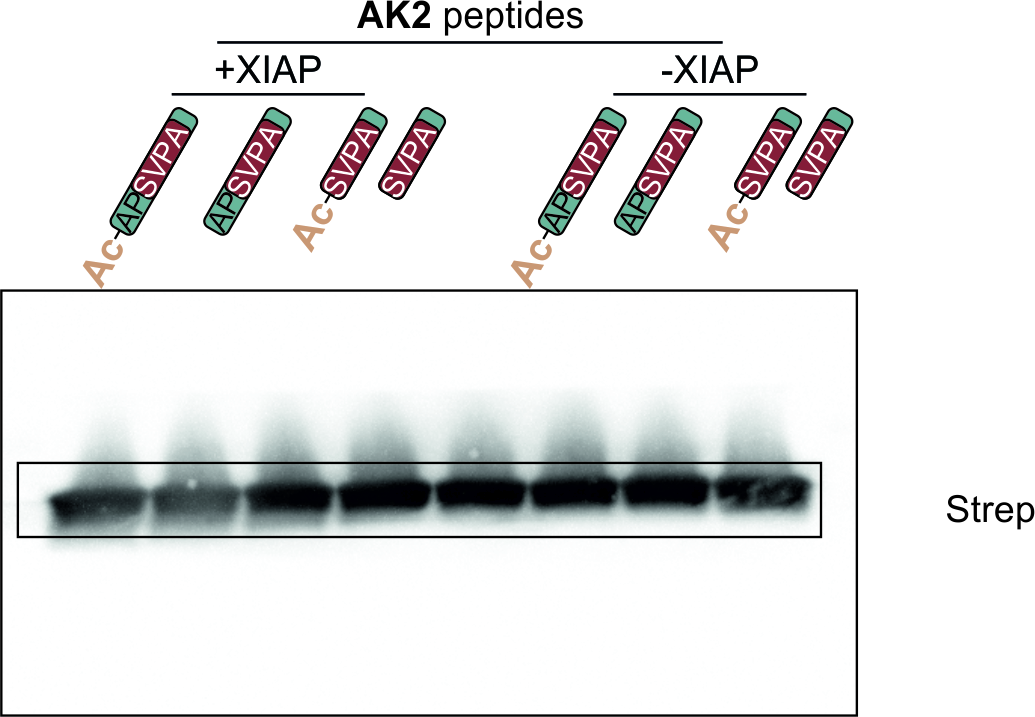

Supplement: Supplementary file 5 — Source data Fig. 3 [file 44319_2025_455_MOESM5_ESM.zip › Figure 3/3I/western Streptavidin.tif]

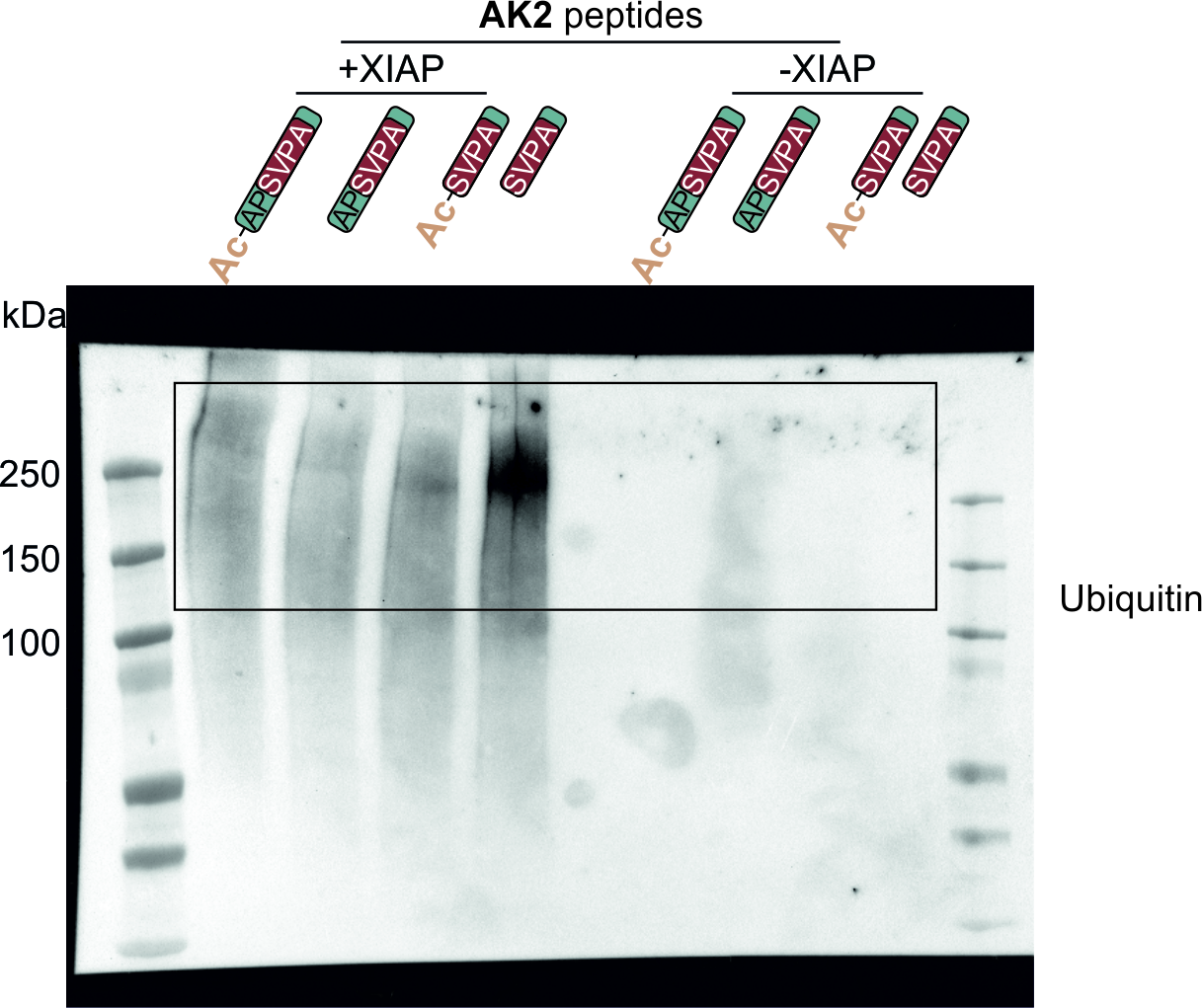

Supplement: Supplementary file 5 — Source data Fig. 3 [file 44319_2025_455_MOESM5_ESM.zip › Figure 3/3I/western Ubiquitin.tif]

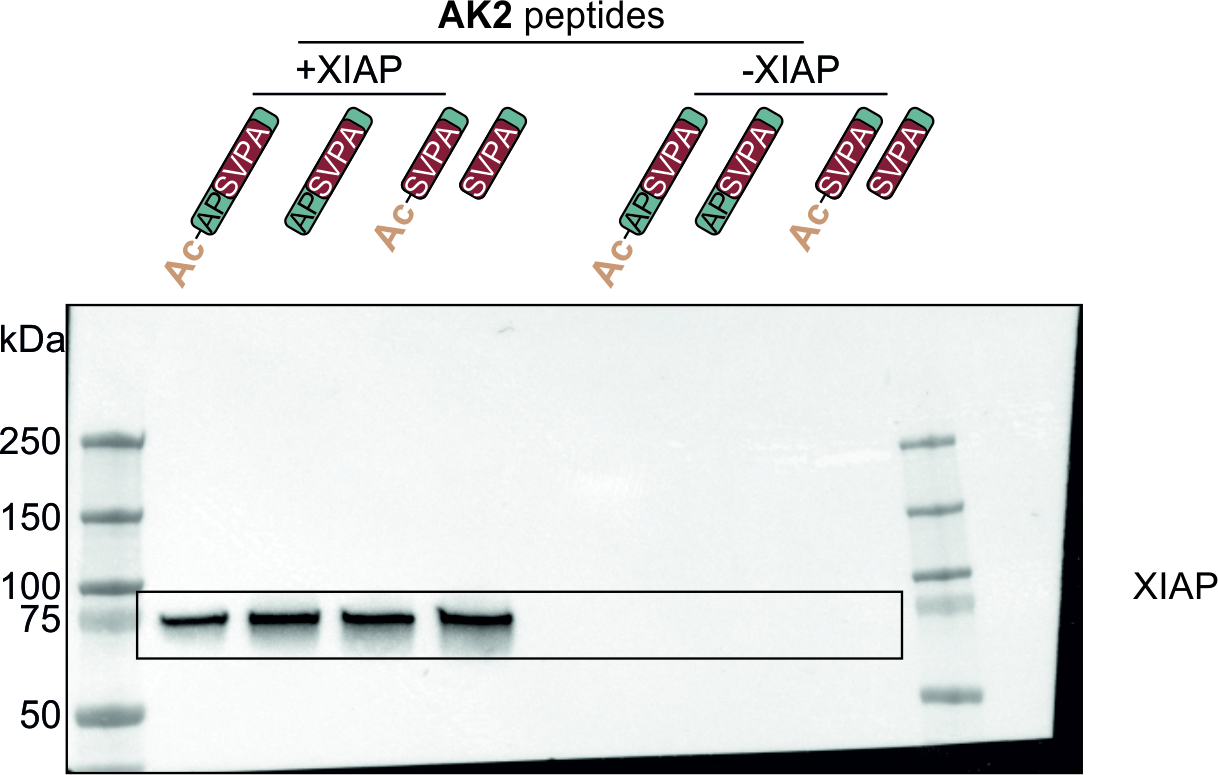

Supplement: Supplementary file 5 — Source data Fig. 3 [file 44319_2025_455_MOESM5_ESM.zip › Figure 3/3I/western XIAP.tif]

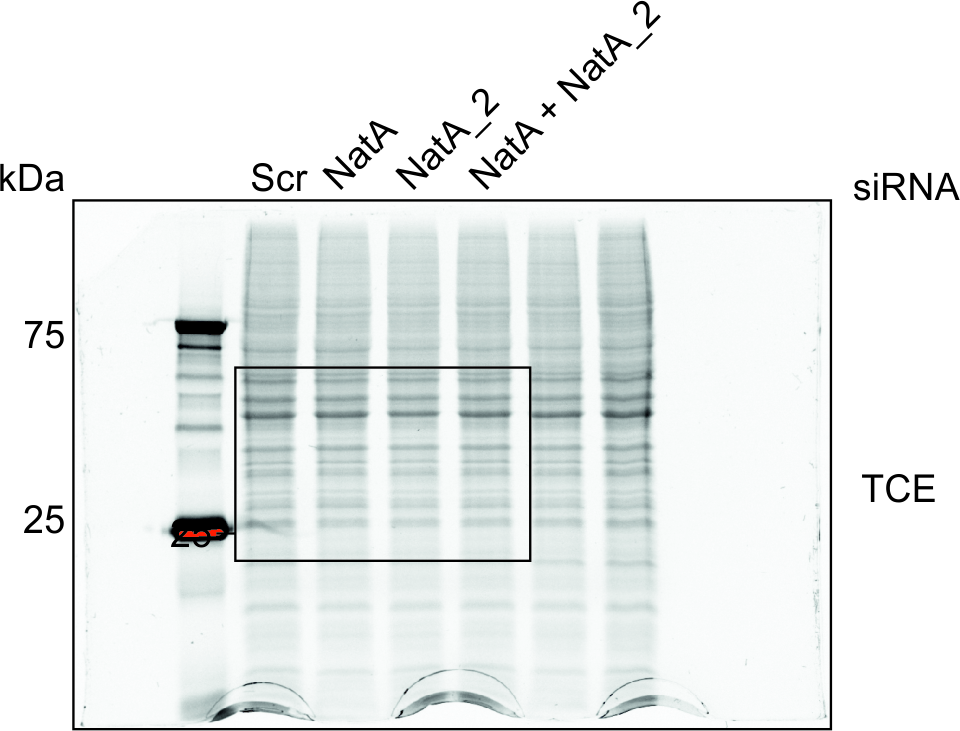

Supplement: Supplementary file 6 — Source data Fig. 4 [file 44319_2025_455_MOESM6_ESM.zip › Figure 4/4I/SDS-PAGE TCE.tif]

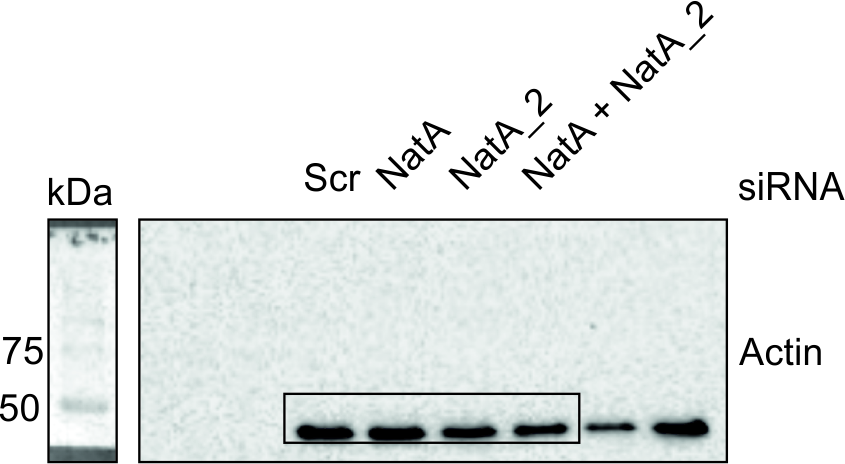

Supplement: Supplementary file 6 — Source data Fig. 4 [file 44319_2025_455_MOESM6_ESM.zip › Figure 4/4I/western actin.tif]

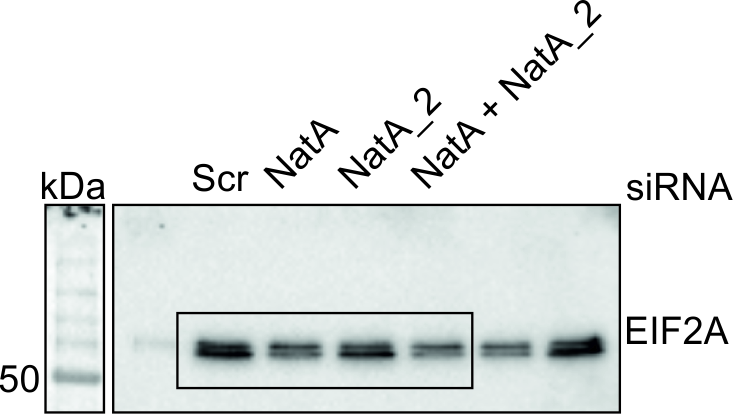

Supplement: Supplementary file 6 — Source data Fig. 4 [file 44319_2025_455_MOESM6_ESM.zip › Figure 4/4I/western EIF2A.tif]

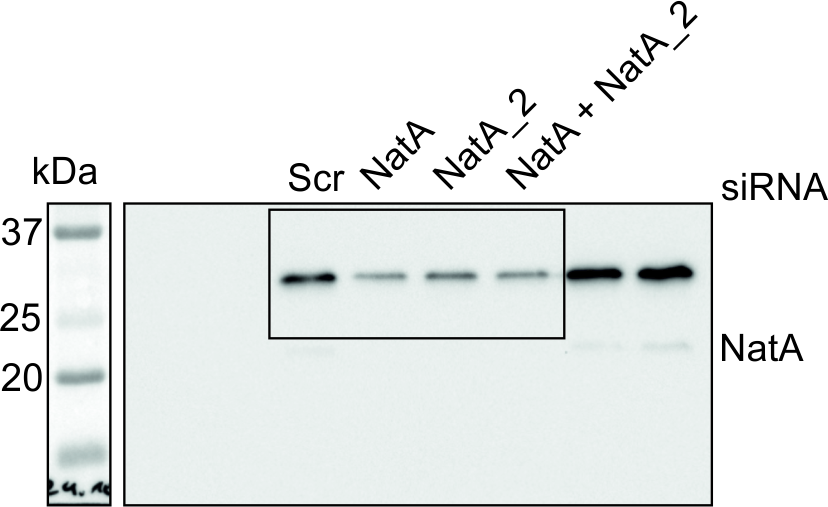

Supplement: Supplementary file 6 — Source data Fig. 4 [file 44319_2025_455_MOESM6_ESM.zip › Figure 4/4I/western NatA.tif]

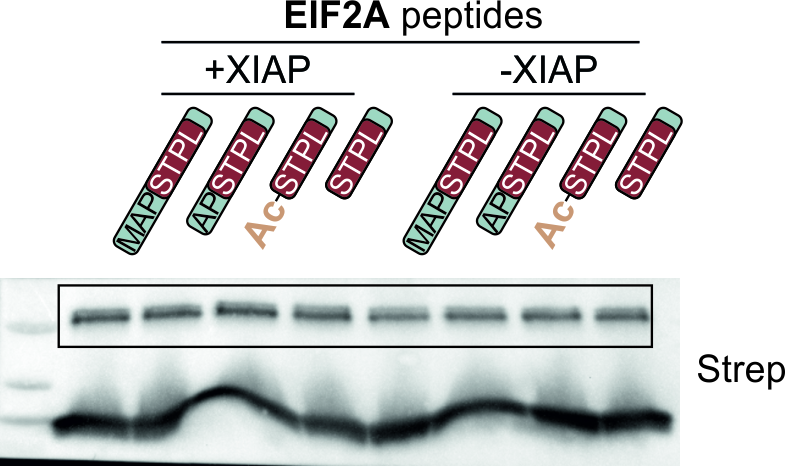

Supplement: Supplementary file 6 — Source data Fig. 4 [file 44319_2025_455_MOESM6_ESM.zip › Figure 4/4J/western Strep.tif]

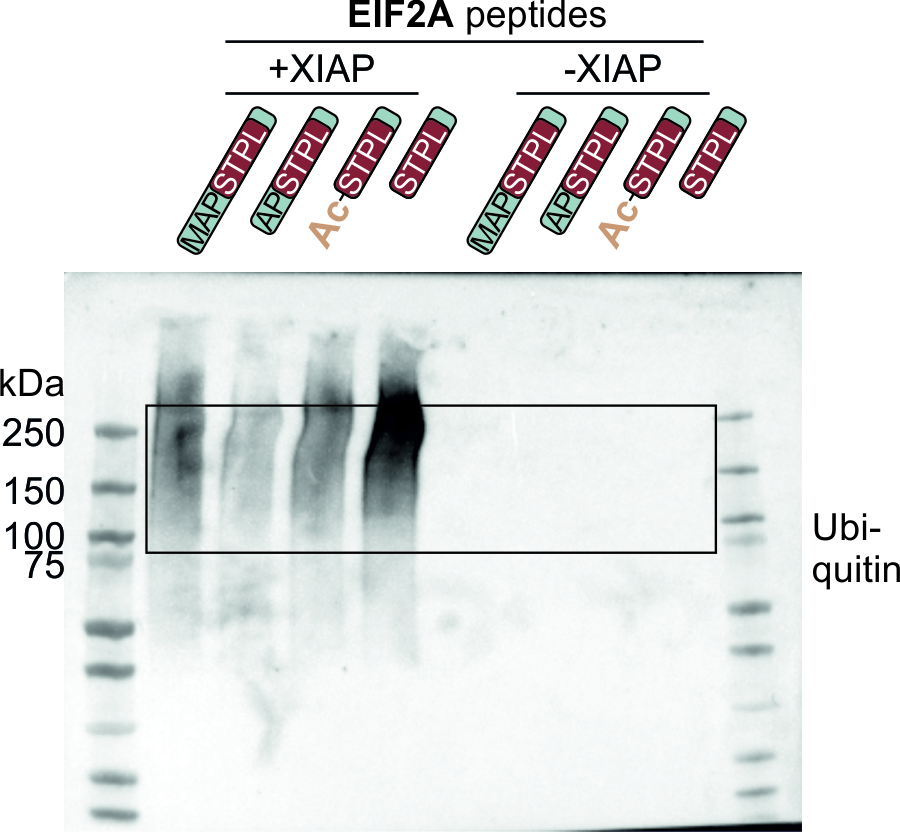

Supplement: Supplementary file 6 — Source data Fig. 4 [file 44319_2025_455_MOESM6_ESM.zip › Figure 4/4J/western Ubiquitin.tif]

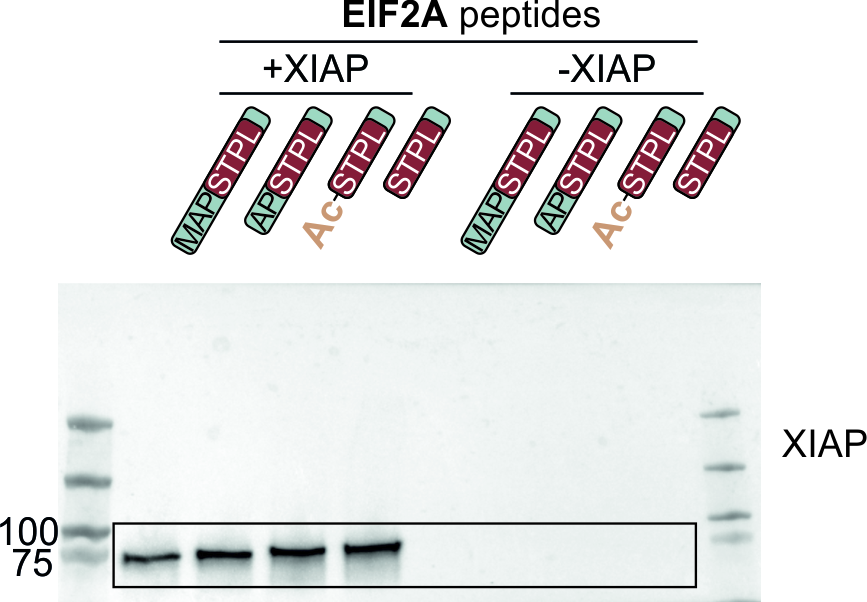

Supplement: Supplementary file 6 — Source data Fig. 4 [file 44319_2025_455_MOESM6_ESM.zip › Figure 4/4J/western XIAP.tif]

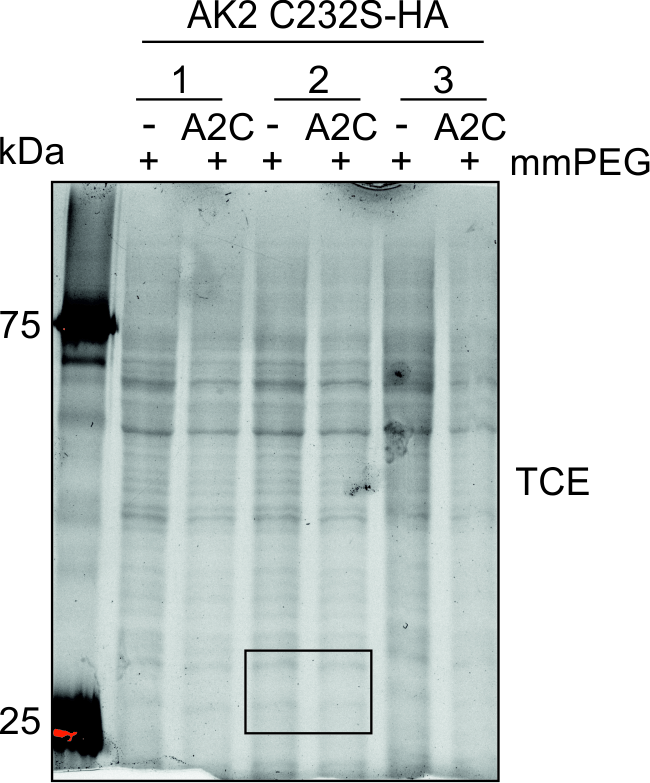

Supplement: Supplementary file 7 — Appendix Figure Source Data [file 44319_2025_455_MOESM7_ESM.zip › Source-Data_SI-figures/Figure S2 (related to Figure 1)/s2c/SDS-PAGE TCE.tif]

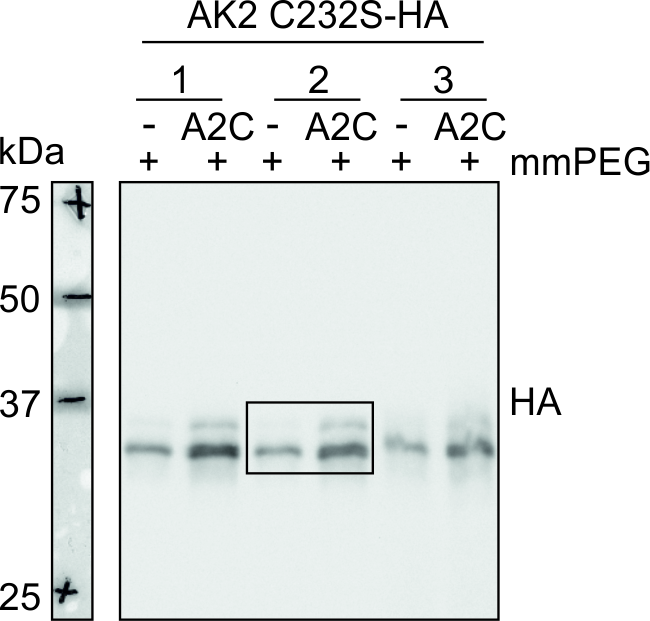

Supplement: Supplementary file 7 — Appendix Figure Source Data [file 44319_2025_455_MOESM7_ESM.zip › Source-Data_SI-figures/Figure S2 (related to Figure 1)/s2c/western HA.tif]

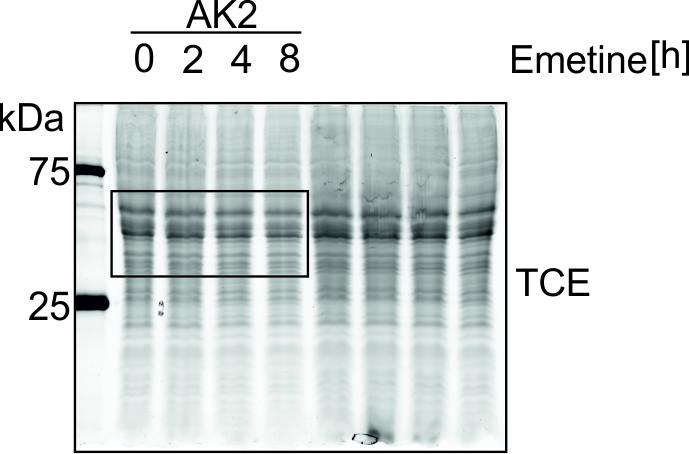

Supplement: Supplementary file 7 — Appendix Figure Source Data [file 44319_2025_455_MOESM7_ESM.zip › Source-Data_SI-figures/Figure S4 (related to Figure 2)/S4B/SDS-PAGE TCE for AK2.tif]

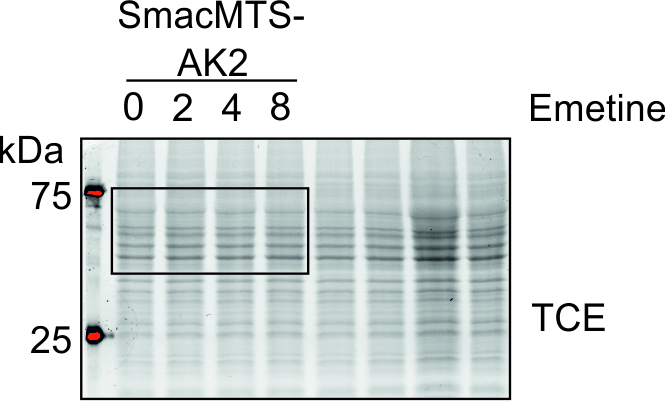

Supplement: Supplementary file 7 — Appendix Figure Source Data [file 44319_2025_455_MOESM7_ESM.zip › Source-Data_SI-figures/Figure S4 (related to Figure 2)/S4B/SDS-PAGE TCE for smacMTSAK2.tif]

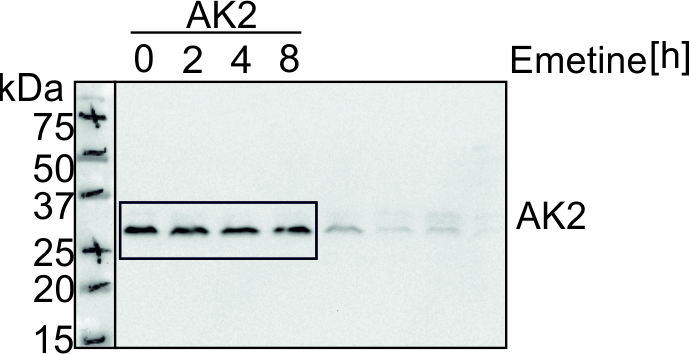

Supplement: Supplementary file 7 — Appendix Figure Source Data [file 44319_2025_455_MOESM7_ESM.zip › Source-Data_SI-figures/Figure S4 (related to Figure 2)/S4B/western AK2 for AK2.tif]

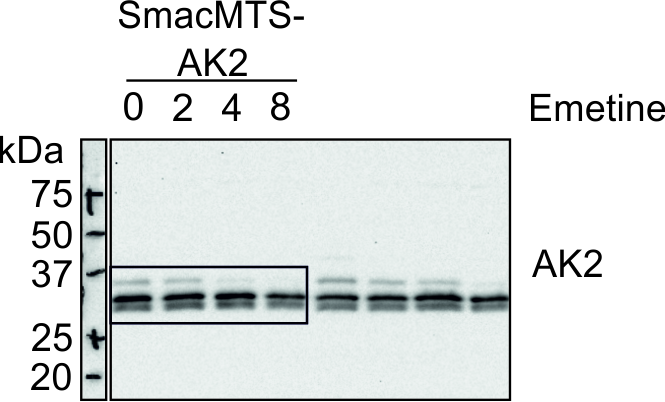

Supplement: Supplementary file 7 — Appendix Figure Source Data [file 44319_2025_455_MOESM7_ESM.zip › Source-Data_SI-figures/Figure S4 (related to Figure 2)/S4B/western AK2 for smacMTS-AK2.tif]

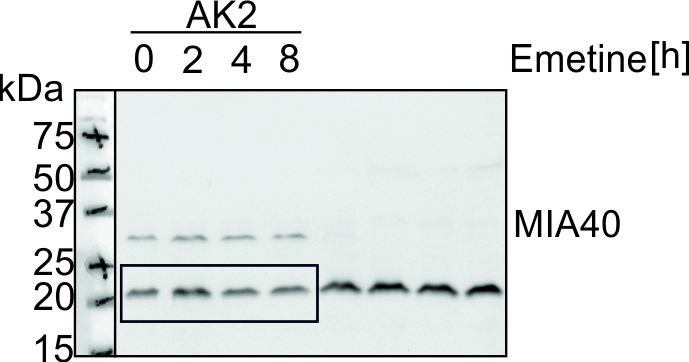

Supplement: Supplementary file 7 — Appendix Figure Source Data [file 44319_2025_455_MOESM7_ESM.zip › Source-Data_SI-figures/Figure S4 (related to Figure 2)/S4B/western Mia40 for AK2.tif]

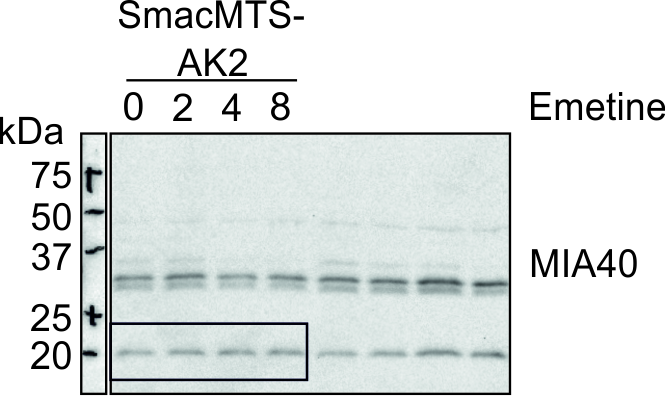

Supplement: Supplementary file 7 — Appendix Figure Source Data [file 44319_2025_455_MOESM7_ESM.zip › Source-Data_SI-figures/Figure S4 (related to Figure 2)/S4B/western Mia40 for smacMTS-AK2.tif]

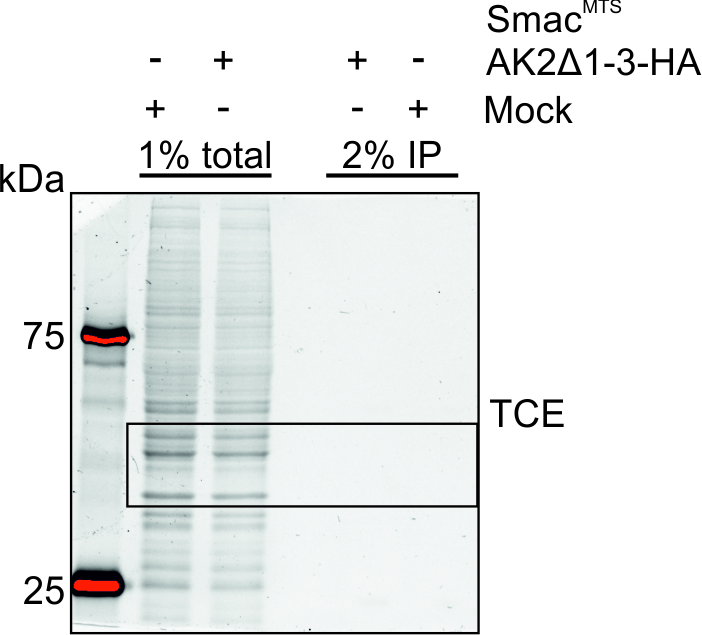

Supplement: Supplementary file 7 — Appendix Figure Source Data [file 44319_2025_455_MOESM7_ESM.zip › Source-Data_SI-figures/Figure S4 (related to Figure 2)/S4C/SDS-PAGE TCE for 2% IP.tif]

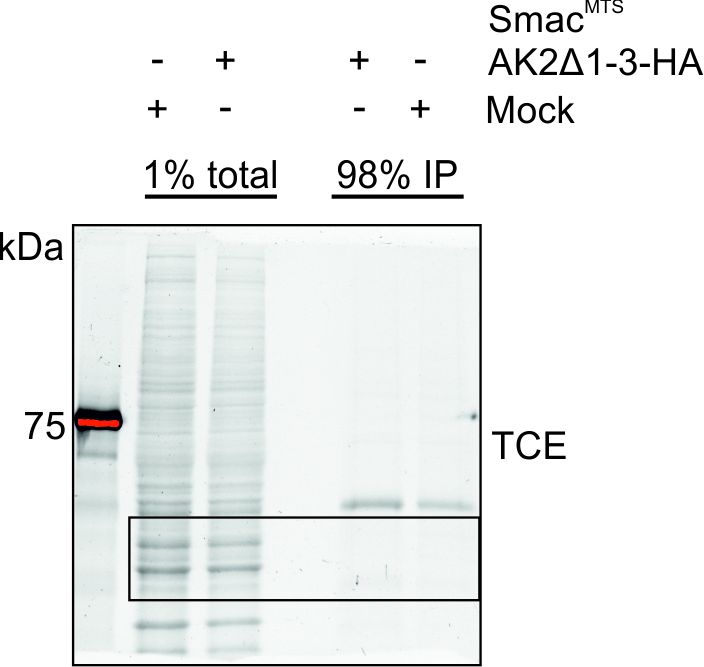

Supplement: Supplementary file 7 — Appendix Figure Source Data [file 44319_2025_455_MOESM7_ESM.zip › Source-Data_SI-figures/Figure S4 (related to Figure 2)/S4C/SDS-PAGE TCE for 98% IP.tif]

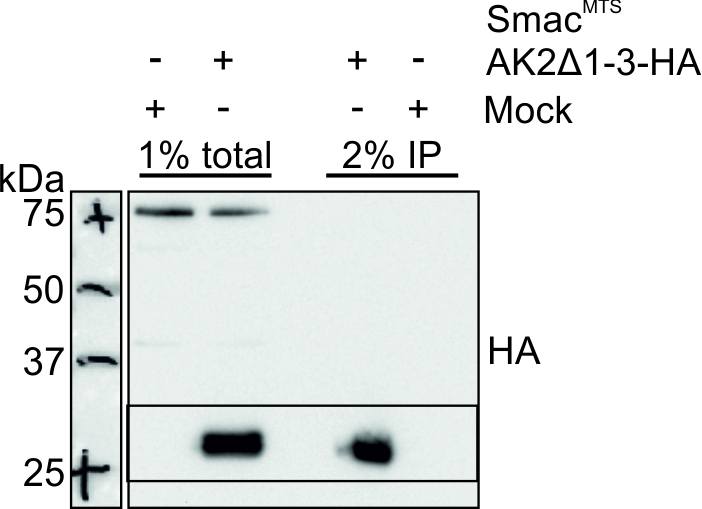

Supplement: Supplementary file 7 — Appendix Figure Source Data [file 44319_2025_455_MOESM7_ESM.zip › Source-Data_SI-figures/Figure S4 (related to Figure 2)/S4C/western HA.tif]

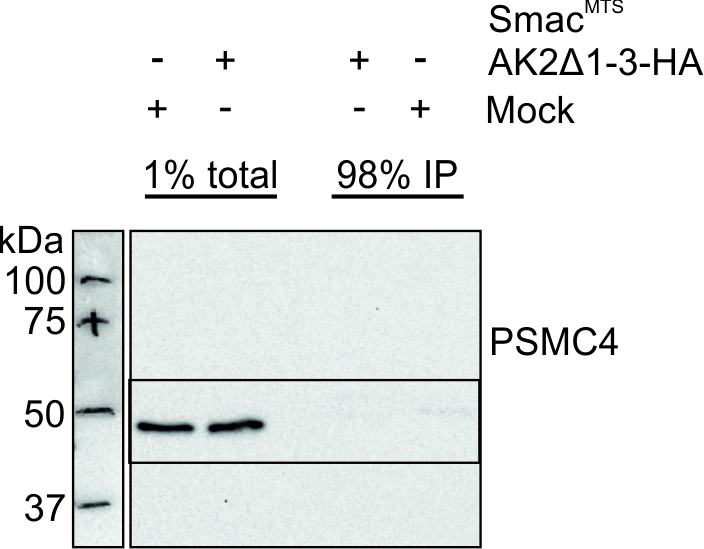

Supplement: Supplementary file 7 — Appendix Figure Source Data [file 44319_2025_455_MOESM7_ESM.zip › Source-Data_SI-figures/Figure S4 (related to Figure 2)/S4C/western PSMC4.tif]

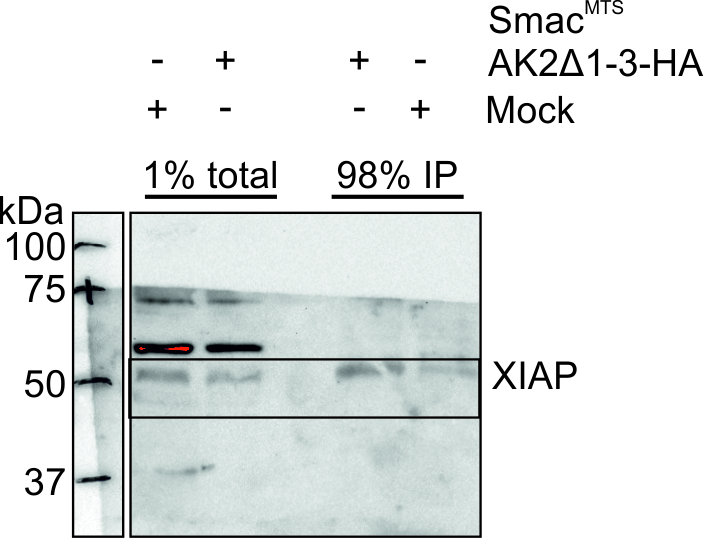

Supplement: Supplementary file 7 — Appendix Figure Source Data [file 44319_2025_455_MOESM7_ESM.zip › Source-Data_SI-figures/Figure S4 (related to Figure 2)/S4C/western XIAP.tif]

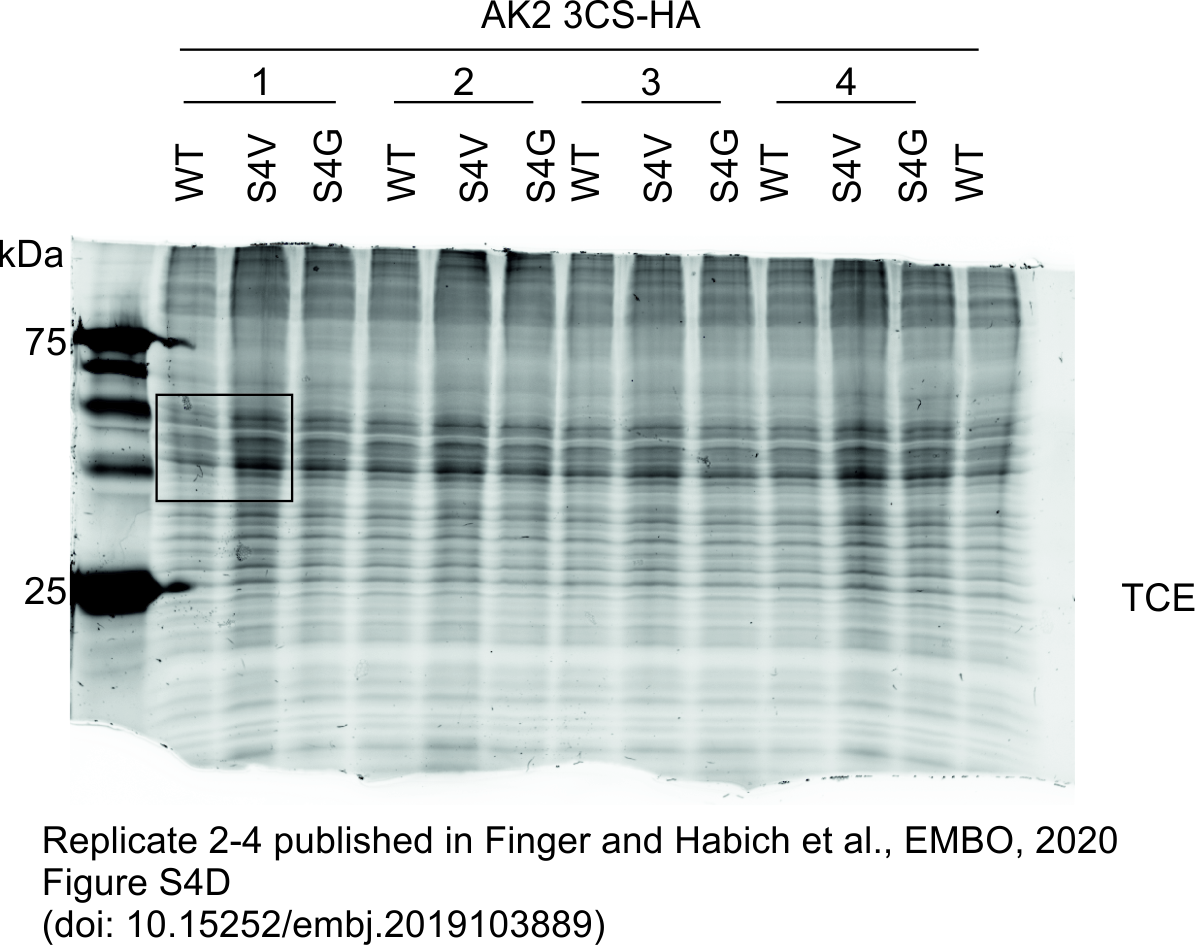

Supplement: Supplementary file 7 — Appendix Figure Source Data [file 44319_2025_455_MOESM7_ESM.zip › Source-Data_SI-figures/Figure S5 (related to Figure 3)/SDS-PAGE TCE.tif]

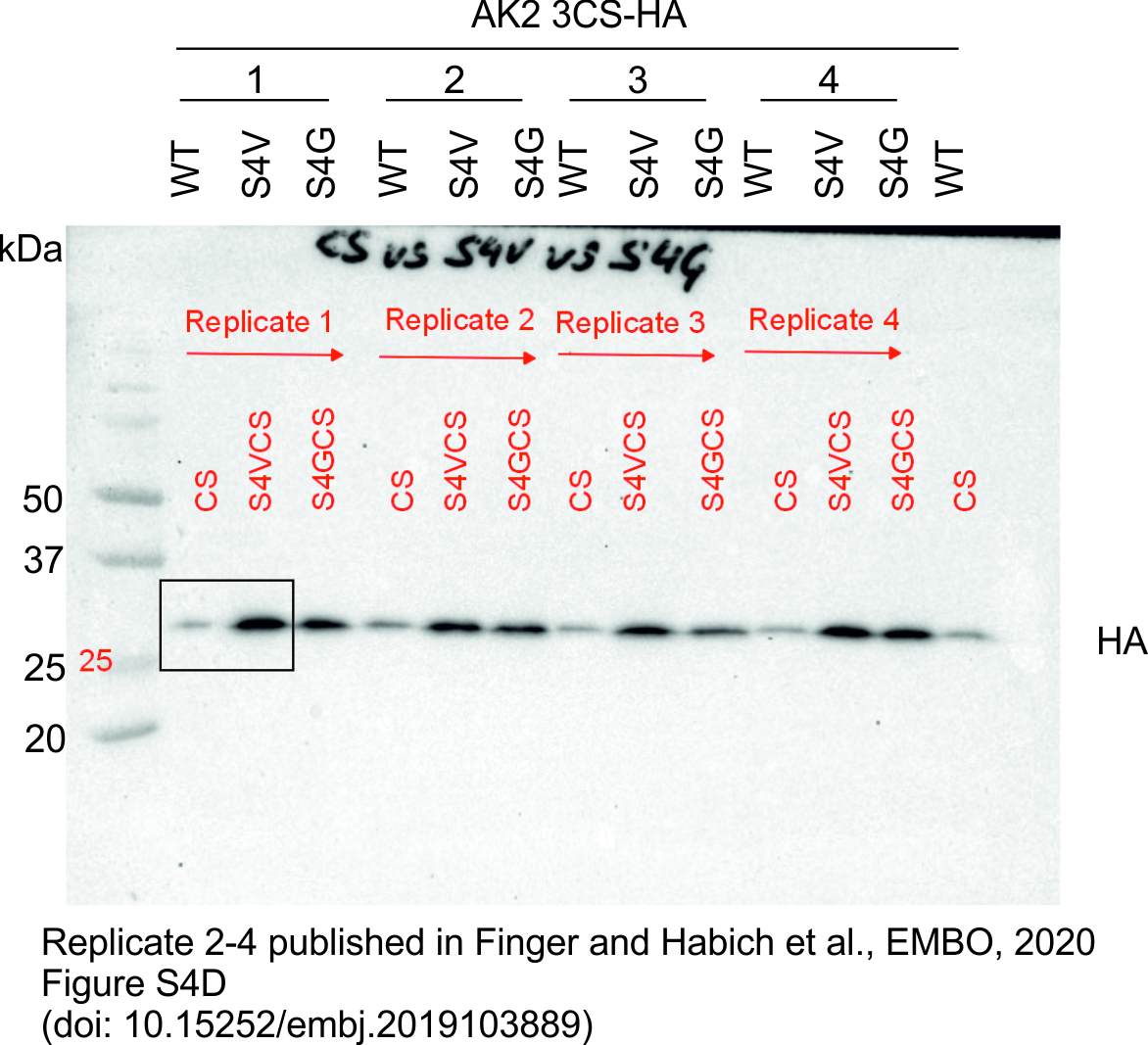

Supplement: Supplementary file 7 — Appendix Figure Source Data [file 44319_2025_455_MOESM7_ESM.zip › Source-Data_SI-figures/Figure S5 (related to Figure 3)/western HA.tif]

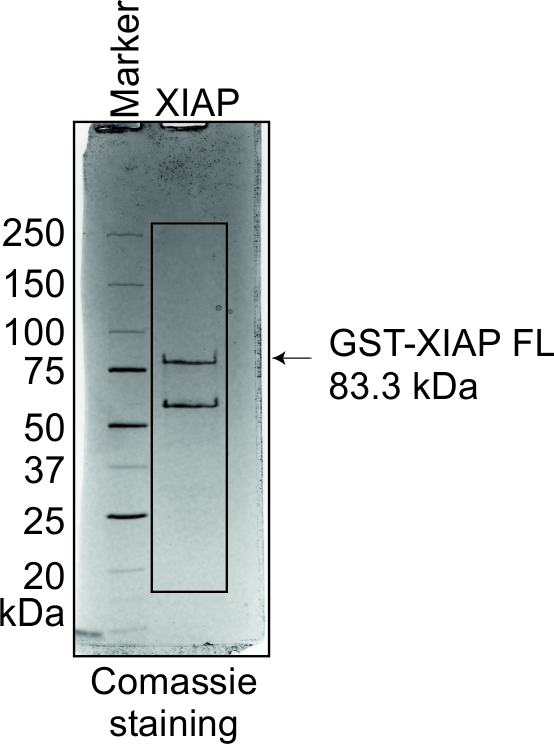

Supplement: Supplementary file 7 — Appendix Figure Source Data [file 44319_2025_455_MOESM7_ESM.zip › Source-Data_SI-figures/Figure S6 (related to Figure 3H-I and Figure 4J)/SDS-PAGE Coomassie.tif]
